# Adding country resolution to EXIOBASE: impacts on land use embodied in trade

**DOI:** 10.1186/s40008-020-0182-y

**Published:** 2020-02-13

**Authors:** Eivind Lekve Bjelle, Johannes Többen, Konstantin Stadler, Thomas Kastner, Michaela C. Theurl, Karl-Heinz Erb, Kjartan-Steen Olsen, Kirsten S. Wiebe, Richard Wood

**Affiliations:** 1 Industrial Ecology Programme, Department of Energy and Process Engineering, Norwegian University of Science and Technology (NTNU), Trondheim, Norway; 2 Institute of Social Ecology, University of Natural Resources and Life Sciences, Vienna (BOKU), Vienna, Austria; 3 Senckenberg Biodiversity and Climate Research Centre (SBIK-F), Frankfurt Am Main, Germany; 4 SINTEF Industry, 7465 Trondheim, Norway

**Keywords:** Multiregional input–output analysis, EXIOBASE, Land use embodied in trade, Country resolution, Rest-of-the-world regions, Regional aggregation, Land footprints

## Abstract

Multiregional input–output (MRIO) databases are used to analyze the impact of resource use and environmental impacts along global supply chains. To accurately account for pressures and impacts that are highly concentrated in specific sectors or regions of the world, such as agricultural and land-use-related impacts, MRIO databases are being fueled by increasingly more detailed data. To date no MRIO database exists which couples a high level of harmonized sector detail with high country resolution. Currently available databases either aggregate minor countries into rest-of-the-world (WIOD and EXIOBASE 3), or the high country resolution is achieved at the cost of non-harmonized or lower sectoral detail (Eora, OECD-ICIO or the GTAP-MRIO). This aggregation can cause potentially significant differences in environmental and socioeconomic impact calculations. In this paper, we describe the development of an EXIOBASE 3 variant that expands regional coverage from 49 regions to 214 countries, while keeping the high and harmonized sectoral detail. We show the relevance of disaggregation for land-use accounting. Previous rest-of-the-world regions supply one-third of global land, which is used to produce a large range of different products under very different levels of productivity. We find that the aggregation of regions leads to a difference in the balance of land embodied in trade of up to 6% and a difference of land embodied in imports of up to 68% for individual countries and up to 600% for land-use-relevant sectors. Whilst the database can still be considered experimental, it is expected to increase the accuracy of estimates for environmental footprint studies of the original EXIOBASE countries, and provides the first estimates for the countries in the previous rest-of-the world.

## Introduction

From the early developments of domestic input–output analysis starting with Leontief (1936), the scope has broadened, both to account for trade relationships across economies (Leontief and Strout 1963) and to extend the framework to enable the attribution of social and environmental impacts, domestic and abroad, to economic activities (Leontief 1970; Miller and Blair 2009). Multiregional input–output (MRIO) models have been widely used in carbon footprint calculations as they provide an appropriate methodological framework for calculations at the national, international and global level (Wiedmann 2009b). In later years, MRIO applications have extended to a wide range of footprint analyses, such as material (Wiedmann et al. 2015; Ivanova et al. 2016; Bruckner et al. 2012; Wiebe et al. 2012), land (Ivanova et al. 2016; Steen-Olsen et al. 2012; Weinzettel et al. 2013), biodiversity (Verones et al. 2017; Lenzen et al. 2012; Wilting et al. 2017, Többen et al. 2018; Marques et al. 2019), labor (Alsamawi et al. 2014a; Simas et al. 2014), income inequality (Alsamawi et al. 2014b) and energy (Wiedmann 2009a; Owen et al. 2017).

The strength of MRIO analysis as a methodology for environmental impact assessment is its ability to trace the impacts of products through the whole supply chain and attribute the impacts at different stages of production to final consumers (Moran and Wood 2014). This enables MRIO analysis to trace increasingly fragmented international supply chains across primary, secondary and tertiary producers, to give a more complete picture of the impacts of final consumption of nations, in comparison to biophysical accounting methods purely based on physical data (Bruckner et al. 2015). A drawback of MRIO analysis in environmental impact studies is the lacking resolution to trace specific products and/or materials (Schaffartzik et al. 2015) or differentiate production technologies in detail. In addition, the efforts to harmonize sectoral and regional data and satellite accounts may require additional aggregation that can compromise the accuracy of environmental and socioeconomic results (Steen-Olsen et al. 2014; Lenzen 2011).

Today several global MRIO databases exist, such as Eora (Lenzen et al. 2013), WIOD (Timmer et al. 2015), GTAP-MRIO (Aguiar et al. 2016), the OECD-ICIO (Yamano and Webb 2018), and EXIOBASE (Tukker et al. 2013). Ideally, a global MRIO is as detailed as possible on both the product/industry resolution as well as on the number of explicitly represented countries. In addition, the ideal MRIO should be available as a consistent long and up-to-date time series and provide detailed socioeconomic and environmental extensions (Tukker and Dietzenbacher 2013). In order to have a consistent database between different world regions, MRIO developers necessarily need to deal with aggregations of extensions, regions and sectors into a standardized classification system (Lenzen 2011). Due to lack of easily available data for many countries, the approach sometimes used to reach global coverage is by estimating “rest-of-the world regions” (RoW), which typically consist of the remaining countries that are not explicitly covered in the database. In EXIOBASE and WIOD, RoW regions comprise over one-third of the world population and 33–44% of global land use, and the aggregation of countries into regions can potentially underestimate impacts embodied in trade, in particular for highly localized pressures such as land use (Stadler et al. 2014).

Discrepancies in environmental impact results across MRIOs are well-documented (Giljum et al. 2019; Owen et al. 2014, 2016; Wieland et al. 2018) and hamper the policy uptake of MRIO results (Moran and Wood 2014; Peters 2007). The robustness of MRIO compared to other methods for estimating sector-specific environmental impacts such as for land use is disputed in the literature. For instance, Schaffartzik et al. (2015) compared biophysical methods and MRIO studies on land use and found a high correlation in regional results for various land use types per capita, except for a few outliers. On the other hand, when trying to interpret MRIO results in comparison to physical trade results, Kastner et al. (2014) found that China is a major net importer of cropland products and embodied cropland in MRIO studies, while physical trade analyses show the opposite. Hubacek and Feng (2016) argue that part of this discrepancy in results between analyses based on MRIO and physical trade balances can be attributed to the differencing system boundaries and conceptual differences, and thus the methods tackle different research questions. Bruckner et al. (2015) summarize the conceptual challenges of using MRIO for attributing land use impacts, especially where aggregation is performed due to lack of product detail (Weinzettel et al. 2014) and regional detail (Stadler et al. 2014). In terms of robustness of impact assessment results from MRIOs, Su et al. (2010) find that around 40 sectors are sufficient to avoid large uncertainties in CO_2_ emissions embodied in exports. Comparing the impacts embodied in exports by disaggregating the SUTs of EXIOBASE at a detail of 59 sectors versus 129 sectors, Wood et al. (2014) found differences in the order of maximum 5% for labor and compensation of employees, while CO_2_ impacts differed up to 50%. Steen-Olsen et al. (2014) further investigated the effect of sector aggregation on CO_2_ multipliers (kg CO^2^/$) in different MRIO databases. Similar to Wood et al. (2014), they found that aggregating sectors of different MRIOs to 17 sectors significantly changed the CO_2_ multipliers, and that the multiplier errors increased with increased sectoral detail in the original database. Similarity in economic input structures among sectors did not imply similarity in terms of emission profiles. This advocates for high sectoral detail despite the potentially much larger compilation effort when building MRIOs. This view is supported by Lenzen (2011) who proposed that aggregating environmental extensions to sectors is a large source of uncertainty as they can be highly heterogeneous. Consequently, Lenzen (2011) proposed disaggregating input–output structures to match the detail of the environmental extensions as the best option for estimating input–output multipliers and reducing uncertainties.

The effects of regional aggregation in MRIOs were studied by Bouwmeester and Oosterhaven (2013). Using EXIOBASE, they find large deviations in regional CO_2_ footprints (up to 22%) and water use (up to 84%) when aggregating 43 regions to four broad regions and one rest-of-the-world region. Su and Ang (2010) find that energy-related CO_2_ emissions are highly dependent on regional aggregation when using an MRIO of China, comparing China as a single region versus split into eight regions. Nevertheless, an earlier paper by Miller and Shao (1990) using an US MRIO model suggests that regional aggregation leads to smaller uncertainties than sectoral aggregation. In part, this is supported by de Koning et al. (2015) who found the aggregation of extensions to be more important than regional and sectoral aggregation for absolute material footprints. Although, due to a significant share of global material extraction in the global south, a more detailed regional coverage of this region in EXIOBASE has been called for by Wiebe et al. (2019). The study of regional aggregation effects due to the RoW aggregation by Stadler et al. (2014) showed that the RoW regions’ share of global land use (33–44% of the global total) are much larger than the equivalent share of global warming potential (17–22%). Furthermore, Stadler et al. (2014) found that 38% of global land exports originate in the RoW regions, underlining the need for a higher country resolution to reduce uncertainties in estimating land use embodied in trade.

In terms of available MRIO databases, EXIOBASE has the highest consistent sector resolution of the available MRIO databases, but is limited in regional resolution. Eora has high country coverage and higher sector detail for some counties, but as the level of detail varies from region to region, this complicates the between-region comparison of impacts on a sectoral level. For example, Eora has only one sector aggregating all agricultural, forestry and fishing activities for most countries in the world. The GTAP-MRIO probably has the best compromise of sectoral resolution (57 sectors) and country (140 regions), but is currently not available as a time series, and has limited sectoral resolution outside the agricultural and food sectors. Ideally, there would be a MRIO database with high sector resolution, individual country coverage and a full time-series.

The aim of this paper is to describe the steps towards such an improved MRIO, by increasing the country resolution of EXIOBASE 3 to explicitly including all domestic economies registered in the UN main aggregates database (214 countries, see below).

We use this extended EXIOBASE (named EXIOBASE 3rx) to show the relevance of additional regional disaggregation to estimate land use embodied in trade. We study the degree of regional aggregation errors on both a regional and on a harmonized and detailed product level.

In the following method section, we describe the development of EXIOBASE 3rx and present its methodological building blocks, describe the processing of land use extensions, and the method for comparing the two databases with different regional resolution. In the result section, we present land footprints and explore the degree of regional aggregation errors for land use embodied in trade. To isolate the effect of regional aggregation on land use, we compare an EXIOBASE version where the MRIO structure is pre-aggregated (aggregation of IO data before calculation of coefficients and results), referred to from now on as the aggregated database, with EXIOBASE 3rx, where the land use results of the full detailed database are aggregated to 49 regions. The implication of this work is further picked up in the next section, where we discuss our results for both MRIO development and the use of MRIO for land use studies now and in the future.

## Methods

### Building EXIOBASE 3rx

**Fig. 1:**
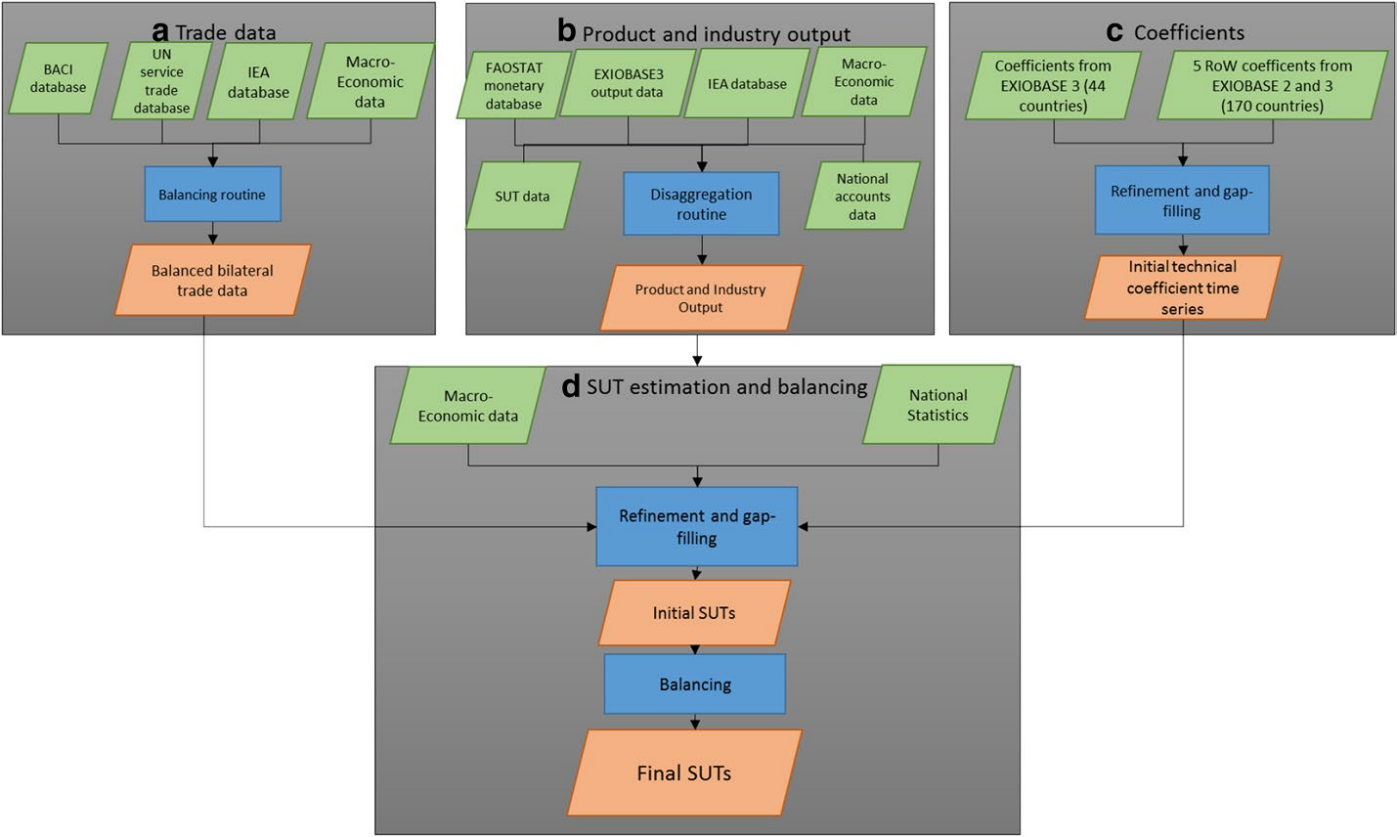
EXIOBASE 3rx: compilation steps for monetary supply use tables. Approach based on figure in Stadler et al. (2018)

The approach to building the monetary supply–use tables for EXIOBASE 3rx (Fig. 1) closely follows previous approaches establishing EXIOBASE 3 and EXIOBASE 2 (Wood et al. 2015, Stadler et al. 2018). Deviations from the EXIOBASE 3 workflow can be found in Additional file 1: S1. In EXIOBASE 3, the economic structures of 44 regions are available in the form of (aggregate) supply–use tables (SUTs). These SUTs are both disaggregated and balanced to product, industry, and trade data. From the SUTs, a trade-linking procedure (Wood et al. 2015) and application of an IO construct (Majeau‐Bettez et al. 2014) is applied to obtain square MRIO tables. In order to estimate the SUTs for the RoW regions in EXIOBASE2 and 3, global average coefficient data was reconciled with product output, industry and trade data (see Stadler et al. (2014) for more information). EXIOBASE 3 adds top-level constraints of macroeconomic data to ensure consistency between regions and over time at a highly aggregate level.

EXIOBASE 3 had a strong European focus (28 EU member states, 16 major economies) and 5 RoW regions (RoW Asia and Pacific, RoW Europe, RoW Africa, RoW America, RoW Middle East). In this work, we extend the procedure used in estimating RoW regions in EXIOBASE 3, but apply it to individual countries in order to expand the number of regions from originally 49 to 214 (Additional file 2). As SUT data are not commonly available for the countries in the RoW regions, we follow the regional approach where we use proxy data in the form of generic estimates of coefficients of the supply (i.e., market share relationships) and use matrices (intermediate use and final demand coefficients) to give an initial estimate of the product/industry transactions. The coefficients are then reconciled to globally balanced estimates of trade data, estimates of product outputs for every country and macroeconomic data on value added, taxes, exports, imports, final consumption and gross capital formation (for an overview of regional data sources, see Additional file 1: S2). The macroeconomic data serve as the top-level data towards which all the other data are balanced. The number of countries is based on the available macroeconomic data from the UN National Main Aggregates Database (United Nations 2018a). Additionally, we estimate land use extensions for all 214 countries (more info in Additional file 1: S11).

### Trade estimates and reconciliation

In order to process the country-specific trade data, we combine data from three data sources when compiling the trade estimates. The BACI database is the main data source (balanced product trade data based on the UN Comtrade database, for more information see Gaulier and Zignago (2010)), while the UN services trade database (United Nations. 2018b) and the IEA database (International Energy Agency 2018) provide data for services and energy products/services, respectively. Re-exports are estimated in the same way as EXIOBASE 2 and 3 (based on SUT data for re-exports where available, and extrapolated based on Comtrade data).

After compiling the initial estimate of the trade data, this is reconciled against the top-level macroeconomic trade data in current price obtained from the UN National Accounts Main Aggregates Database. Here, we replace the quadratic programming approach with an information theoretical approach. We minimize cross-entropy (CE), also known as Kullback–Leibler Divergence (Kullback and Leibler 1951), between the final trade flows of product $i$ from country $r$ to country $s$, $p_{i}^{rs}$, and their initial estimate $q_{i}^{rs} ,$ subject to constraints requiring that total export and import values from the UN National Main Aggregates Database, $EX^{r}$ and $IM^{s}$, are met. In addition to the constraint that total exports by country and product are less than gross output, $x_{{{ \text{max} }_{\text{i}} }}^{r}$. For the general methodology, see Golan and Vogel (2000). As in Többen and Schröder (2018), we implement the computationally much more efficient unconstrained dual of the minimal cross-entropy problem. In the dual version, the cross-entropy model takes the form

1 $$\begin{aligned} \mathop {\text{max} }\limits_{\lambda } D = & \mathop \sum \limits^{r} \lambda_{1}^{r} EX^{r} + \mathop \sum \limits^{s} \lambda_{2}^{s} IM^{s} + \mathop \sum \limits_{i}^{r} \left( {\lambda_{{{ \text{max} }_{i} }}^{r} - \lambda_{{{ \text{min} }_{i} }}^{r} x_{{{ \text{max} }_{i} }}^{r} } \right) \\ & \quad - \mathop \sum \limits_{i}^{rs} q_{i}^{rs} \exp \left\{ {\lambda_{1}^{r} + \lambda_{2}^{s} + \lambda_{{{ \text{max} }_{i} }}^{r} - \lambda_{{{ \text{min} }_{i} }}^{r} } \right\} \\ & \quad - \mathop \sum \limits_{i}^{r} \lambda_{{{ \text{max} }_{i} }}^{r} x_{{{ \text{max} }_{i} }}^{r} - \mathop \sum \limits_{i}^{r} \lambda_{{{ \text{min} }_{i} }}^{r} x_{{{ \text{min} }_{i} }}^{r} , \\ \end{aligned}$$

where $\lambda_{1}^{r}$ and $\lambda_{2}^{s}$ are Lagrangian multipliers referring to the equality constraints. Following the approach of Kazama and Tsujii (2005), the inequality constraints are formulated as lower and upper bounds with $\lambda_{{{ \text{max} }_{i} }}^{r}$ and $\lambda_{{{ \text{min} }_{i} }}^{r}$ being the Lagrangians and $x_{{{ \text{max} }_{i} }}^{r}$ and $x_{{{ \text{min} }_{i} }}^{r}$ being the bounds. In this application, the lower bounds are equal to zero, whereas the upper bounds are equal to gross output by country and product.

From the Lagrangians maximizing $D$, the final trade flows can be computed by

2 $$p_{i}^{rs} = \frac{{q_{i}^{rs} \exp \left\{ {\lambda_{1}^{r} + \lambda_{2}^{s} + \lambda_{{{ \text{max} }_{i} }}^{r} - \lambda_{{{ \text{min} }_{i} }}^{r} } \right\}}}{{\mathop \sum \nolimits_{i}^{rs} q_{i}^{rs} \exp \left\{ {\lambda_{1}^{r} + \lambda_{2}^{s} + \lambda_{{{ \text{max} }_{i} }}^{r} - \lambda_{{{ \text{min} }_{i} }}^{r} } \right\}}}.$$

### Estimating product output

Product output estimates were processed in EXIOBASE 3 (Stadler et al. 2018) and combines data from several national account databases, FAOSTAT (2014), IEA energy balances (IEA 2015) and product output from EXIOBASE 2 (for more information see Additional file 1: S1 and S9 in Stadler et al. (2018)). The main difference is that for EXIOBASE 3rx we process the raw data on an individual country level also for all former RoW countries. In the next step, these data sources served to disaggregate the UN macroeconomic industry output data (United Nations. 2018a), which consists of gross value added from seven aggregated industries. By applying a concordance matrix between the seven UN industries and the 163 EXIOBASE industries (Additional file 1: S3) and by assigning a quality index to the different data sources based on their closeness to raw data, the routine disaggregates the UN industry data. The disaggregation is based on the values in the chosen raw data source. The result is product output at the level of the 163 industries and 200 products of EXIOBASE. In general, this procedure should give reasonable estimates for agricultural, food and energy products, whilst missing detailed country-specific data on manufactured products and services.

### Initial estimates of the input–output structure

For the 44 countries that exist in EXIOBASE 3, the coefficients are used directly as initial estimates in EXIOBASE 3rx. For each of the 170 RoW countries, we use the coefficients from the respective RoW region from EXIOBASE 3. If EXIOBASE 3 coefficients caused balancing problems—such as conflicting constraints between the initial estimate of the SUT and the top-level macroeconomic data, we used EXIOBASE 2 coefficients instead.

### Balancing supply–use tables

The monetary SUT balancing routine applies an algorithm similar to the approach in Stadler et al. (2018) using a quadratic programming target function. One important difference here is that, due to lack of data on a detailed country level, taxes, trade and transport margins are not estimated as explicit layers in our approach. Hence, our system is an MRIO in basic pricing only. The results are monetary SUTs estimated for every country and year independently for a time series from 1995 to 2015 for 214 countries. The balancing routine was unable to find a solution for a few countries, about 3.3% of all cases through the time series. See an overview in Additional file 1: S5 of the unbalanced countries.

### Converting from monetary SUTs to IO tables

To go from individual SUTs to analytical IOTs, we stop at the step before creating fully detailed multiregional input–output tables (see Peters et al. 2011), and instead aim for trade-linked IOTs. This gives us the possibility to apply bilateral trade approaches rather than full MRIO approaches (Peters 2008, and see below). Due to the approach outlined above (balancing trade first, and not changing it in the SUT balancing), we ensure that the final SUTs are globally consistent (i.e., that imports and exports match for trading partners). The result is hence a fully trade-linked SUT system. In the final step, SUTs were converted to IO tables using the procedure described in EUROSTAT (2008). The industry technology construct is applied to deal with co-production. Using this approach, we avoid the problem of negative coefficients that could be faced when applying, e.g., the commodity technology construct (Jansen and Raa 1990). The choice of producing trade-linked IO tables rather than fully compiled MRIO tables (as per EXIOBASE3) was due to the significantly lower loading and running time, and does not constitute a loss of data (we had no additional data to inform the trade relationships). Normal desktop computers are not able to handle the memory requirements of a fully complied MRIO system of the size of EXIOBASE 3rx, but can easily handle the trade-linked system. Because of the trade proportionality assumption over the import use estimates, if a full MRIO system is desired, either the approach of Peters et al. (2011) could be followed if no memory constraints exist, or topological transformation of the data could be applied as explained in Rodrigues et al. (2016).

### Compiling the land use data

To obtain land use data at the sectoral resolution of EXIOBASE, we followed a two-step procedure: First, we created spatially explicit maps for major land cover types based on publicly available state-of-the-art datasets. The data were harmonized following a closed-budget mapping approach (Erb et al. 2007), i.e., the sum of all layers will add up to 100% or the available land area for each specific grid cell. In a second step, we utilized information from census statistics (FAOSTAT) to further disaggregate the data to closely match the EXIOBASE sector classification (in table format). See Additional file 1: S11 for a detailed description of establishing the land use dataset.

The land use extensions comprise 207 countries, which cover most of the countries in EXIOBASE 3rx. For the remaining seven countries, mainly Island states like Palau and Nauru, we use the land area variable from FAOSTAT (2019) to estimate the land use accounts of the missing countries. We first choose a country (country A) with existing land use data and geographical proximity to the country with missing data (country B). Next, the land use extensions of country B are estimated by scaling the data of country A based on the land area variable of country B relative to that of country A. Next, we remap the land use data into EXIOBASE 3rx format. Here, we follow the same procedure as in EXIOBASE 3, and therefore refer the reader to S6 of Stadler et al. (2018). The resulting 40 land use extensions consist of land used by the EXIOBASE 3rx production sectors (**F**) and land directly allocated to households (**F_hh**).

### Estimating land footprints

Due to the large size of EXIOBASE 3rx (e.g., the coefficient matrix (**A**) has 42,800 × 42,800 data points), most of the arrays are saved in a sparse format in MATLAB to reduce disk storage requirements. The sparse format database for one year is approximately 60 megabytes.

We used the emissions embodied in bilateral trade (EEBT) approach (Peters 2007, 2008) to do land use calculations using EXIOBASE 3rx rather than calculating impacts from the MRIO system directly. The main difference is that we do not account for intermediate demand of imports that go to industries to produce exports. Hence, a limitation is that imports that are used for intermediate production, that later end up as exported goods are not accounted for. However, as we are studying aggregate land embodied in trade, and not that resulting from a particular final demand, the EEBT approach is suitable as discussed in Peters (2007). The basic principles of the EEBT approach are explained in S12. Stadler et al. (2014)’s additional information explains the EEBT approach in detail.

### Analyzing the effect of regional aggregation

To enable comparison of the pre-aggregated database and EXIOBASE 3rx for land use results, we aggregate the inter-industry flow matrix (**Z**), the final demand matrix (**Y**), the total land use of production (**F**), and land directly allocated to households (**F_hh**) to 49 regions using a regional bridging (Additional file 2). Next, we calculate the coefficient matrix (**A**) and the land use multipliers (**S)** per monetary unit. We refer to this as the aggregated database from now on. Note that we do not compare land use results of EXIOBASE 3rx and EXIOBASE 3 directly as it would be difficult to distinguish the effect of regional disaggregation to effects arising from other changes (see Additional file 1: S1 for an overview of the differences in workflows between the databases). Two of the most prominent changes to the workflow are the mentioned updated trade processing and reconciliation, and re-processed and more detailed land use extensions. In addition, the land use dataset was newly established specifically for EXIOBASE 3rx.

For comparing the land embodied in trade between the EXIOBASE 3rx and the aggregated database, we define the aggregation error as the sum of the absolute difference of the traded land in question:

3 $$\in = \mathop \sum \limits_{s = 1}^{n} \left( {\left| {T_{{q,r,p_{\text{EXIO3rx}} }} - T_{{q,r,p_{\text{Agg}} }} } \right|} \right) ,$$

where $T$ is a three-dimensional array of land embodied in imports or exports with dimensions imports/exports ($q$) by trade partner ($r$) by product ($p$). $s$ corresponds to the summed-over dimension(s) and $n$ is the number of data points in the summed-over dimension(s). $n$ varies according to the type of aggregation error in question. We examine aggregation errors of imports and exports of products, between regions, and specific product–region combinations. Hence, for, e.g., the product aggregation error of imports, we sum over $q,r$—exporting and importing countries. Similarly, for the aggregation error of exports of specific goods originating in specific countries, we sum over $r$—importing countries. Note that we exclude intra-RoW trade in EXIOBASE 3rx aggregated to 49 regions for the sake of comparison with the aggregated database, where intra-RoW trade is part of domestic demand.

“Aggregation error” refers to the difference in results between those from one input–output table and those from a pure aggregation of the same input–output table prior to calculations (as per literature, e.g., Gibbons et al. (1982)). It must be noted that input–output tables are always estimates of actual transactions and the more disaggregated an input–output table is (especially in the case at hand where there is very poor statistical coverage of some countries) the higher the level of uncertainty of these transactions. Most literature (e.g., Lenzen (2011)) point to the benefit of disaggregation for reducing the uncertainty of footprint calculations, but we do not explore that here. As such, it must be remembered that uncertainty related to disaggregation, and the concept of aggregation error are related, but different concepts. We expect, but cannot measure whether the accuracy of our results will increase by disaggregating EXIOBASE3, whilst we can measure the aggregation error between the disaggregated database and a pure of aggregation of the same database.

Using Eq. 3 we define the aggregation error score $\in_{s}$ as the aggregation error divided by the export/imports of the region, product or product–region combination in the 49 region version of EXIOBASE 3rx:

4 $$\in_{s} = \frac{ \in }{{T_{{q,r,p_{\text{EXIO3rx}} }} }}.$$

## Results

The results of the construction process for EXIOBASE 3rx are available at 10.5281/zenodo.2654460. Country SUTs are available as well as IOTs and land extensions. Furthermore, in Additional file 3 we provide compiled production, consumption and trade-related results for land use. Here, we proceed with an analysis of these results, and the differences introduced by regional disaggregation.

### Trade comparisons

The added regional detail changes the trade structure of EXIOBASE 3rx compared to the aggregated database and EXIOBASE 3. In EXIOBASE 3, intra-RoW trade flows are treated as “domestic” flows, while they are treated as inter-country trade flows in EXIOBASE 3rx. In 2015 intra-RoW trade (as classified in EXIOBASE 3) is the largest or second largest export destination of each continental region (Table 1).

**Table 1:** Percentage of intra-RoW region exports for year 2015

% of exports within region		Rank export partners
RoW Asia and Pacific	22.2	1
RoW Europe	8.6	2
RoW Middle East	15.4	1
RoW America	26.2	1
RoW Africa	11.9	2

This has relevance to the regional disaggregation of EXIOBASE 3 for footprint analyses both for the countries within the RoW region and for the trade partners importing from the RoW region. In the former case a footprint resulting from a demand for an imported good from, e.g., Thailand to the Philippines would be treated as domestic in EXIOBASE 3 with the land use (or emission) intensity equal to the RoW region, while in EXIOBASE 3rx the footprint is treated as imports using the land use intensity of Thailand, which can lead to highly differing results as discussed in the introduction. In the latter case, a final demand of imports from a RoW region with destination in a region outside the RoW region will in both EXIOBASE 3 and EXIOBASE 3rx be treated as an import, but the emission intensity will differ. In EXIOBASE 3 the RoW land use intensity of production is used, while in EXIOBASE 3rx the land use intensity of production of the region now disaggregated from the RoW region forms the basis of the footprint.

### Land footprints

**Fig. 2:**
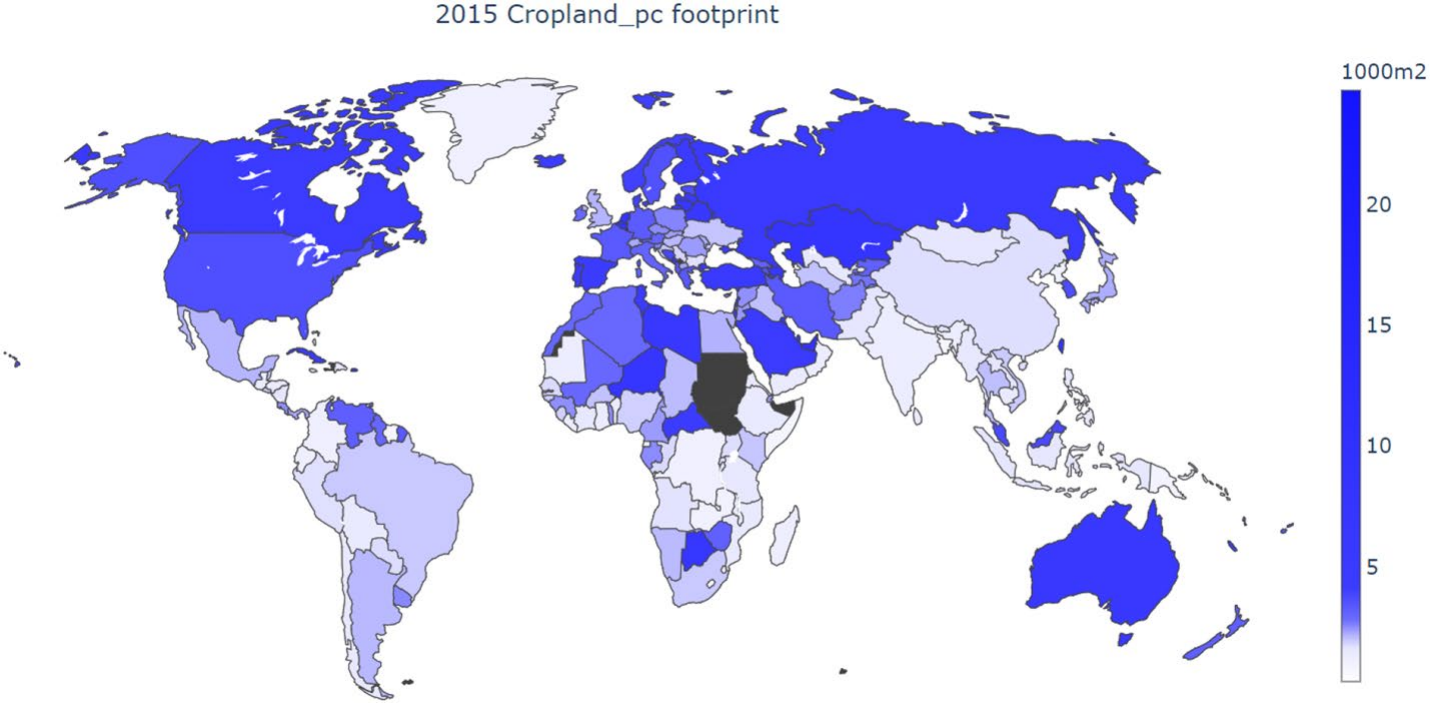
Map of cropland footprints per capita for year 2015 for 214 countries. Unbalanced countries in dark gray (Comoros, Haiti, Liechtenstein, South Sudan and Sudan)

The cropland footprints per capita for all 214 regions in 2015 are presented in Fig. 2 (see Additional file 1: S10 for figures on other land use types and Additional file 3 for per capita footprints for individual land use types and aggregated across all land use types). Monaco has the largest cropland footprint per capita (24,700 m^2^/cap) followed by Luxembourg (19,100 m^2^/cap) and the United Arab Emirates (9 100 m^2^/cap). The lowest footprints are found in Timor-Leste (257 m^2^/cap), Bermuda (336 m^2^/cap), and Zanzibar (353 m^2^/cap). Large economies such as the United States (3620 m^2^/cap), Russia (5250 m^2^/cap), Germany (3260 m^2^/cap) and France (3330 m^2^/cap) have cropland footprints per capita well above the global average of 2130 m^2^/cap, while those of China (1710 m^2^/cap) and India (1260 m^2^/cap) are below the global average. In general, the highest per capita footprints are in Europe, the Middle East, Eastern and Northwestern parts of Asia and a few scattered African countries. The import share of total cropland consumed highly varies between countries (see Additional file 3). With countries in the Middle East, some island states and Eastern parts of Asia, having import shares of 100%, while particularly several African countries import less than 5% of the land area needed to satisfy their cropland consumption. For EXIOBASE 3rx, the global import share of cropland consumption increased from 20.9% in 1995 to 42.7% in 2015.

The global consumption-based per capita forest footprint is 3650 m^2^, with the largest values found for Finland (68,100 m^2^) and New Caledonia (49,300 m^2^), and smallest for Palestine (82.4 m^2^) and Yemen (146 m^2^). The global imported share of total forest consumption is 36.0%. The global per capita grazing land footprint is 3650 m^2^ with an import share of 21.3%. Mongolia (1,34,000 m^2^) and Botswana (97,500 m^2^) have the highest values and North Korea (99.5 m^2^) and Bangladesh (113 m^2^) have the lowest per capita values. The British Virgin Islands (1650 m^2^) and Australia (1500 m^2^) have the highest per capita infrastructure footprints, well above the global average of 185 m^2^. The total land use summed across all land types has grown by 1.6% from 1995 to 2015. On a per capita basis, global land use has decreased from 15 600 m^2^ ha/capita to 12 300 m^2^/capita (27%) from 1995 to 2015. This is driven by a moderate decrease in consumption-based land use in populous countries such as India, Brazil and the United States, and a stronger decrease in several African countries. Increases in countries such as China, Germany and the Netherlands partly offset the effect.

Overall there is a factor of 2.20 increase of land embodied in trade from 1995 to 2015. This increase is driven by a growth in exports from geographically large countries such as Russia, Australia and Brazil. China has largely single-handedly driven the global increase in imported land, from 2.3% of the global total in 1995 to 27.4% in 2015. At the same time, the global share of imported land has decreased particularly for Japan (9.5% in 1995 and 3.6% in 2015) and the United States (11.5% in 1995 and 8.4% in 2015).

### Comparison of regional disaggregation

EXIOBASE 3rx shows global land embodied in trade as 25.8% of global land use, compared to 24.2% in the aggregated database (Table 2) (For equivalent results for all countries in EXIOBASE 3rx, see Additional file 1: S13.) Comparing country-specific trade balances of land for the databases, there is consistency in which countries are net importers and exporters, but there is a difference of up to 5.9% in the balance of land embodied in trade between the databases.

**Table 2:** Land area use from production, consumption, exports as share of production, imports as share of consumption, and the balance of land area embodied in trade (BLET) for EXIOBASE 3rx aggregated to 49 regions and the aggregated database for year 2015 (adapted from Peters and Hertwich 2008)

Region	EXIOBASE 3RX					Aggregated database				
	Production (km^2^)	Consumption (km^2^)	Exports %	Imports %	BLET %	Consumption (km^2^)	Exports %	Imports %	BLET %	BLET difference
Austria	80,300	119,000	48.6	65.4	− 16.8	120,000	48.6	65.7	− 17.0	0.2
Belgium	30,600	227,000	66.6	95.5	− 28.9	199,000	66.6	94.9	− 28.3	− 0.6
Bulgaria	110,000	75,100	44.7	19.4	25.3	76,300	44.7	20.6	24.1	1.2
Cyprus	9000	9980	38.6	44.7	− 6.0	10,300	38.6	46.1	− 7.5	1.5
Czech Republic	78,800	88,000	49.1	54.5	− 5.4	88,800	49.1	54.9	− 5.8	0.4
Germany	355,000	796,000	37.3	72.0	− 34.7	813,000	37.3	72.6	− 35.3	0.6
Denmark	43,300	72,300	56.0	73.6	− 17.6	73,500	56.0	74.1	− 18.0	0.4
Estonia	43,200	21,300	77.3	53.9	23.3	21,100	77.3	53.5	23.8	− 0.4
Spain	499,000	517,000	39.9	42.0	− 2.1	545,000	39.9	44.9	− 5.0	2.9
Finland	284,000	467,000	35.5	60.7	− 25.2	466,000	35.5	60.7	− 25.1	− 0.1
France	588,000	837,000	32.8	52.8	− 20.0	815,000	32.8	51.5	− 18.8	− 1.2
Greece	126,000	134,000	32.6	36.4	− 3.7	133,000	32.6	36.0	− 3.4	− 0.3
Croatia	54,800	53,700	20.2	18.6	1.6	54,400	20.2	19.7	0.6	1.0
Hungary	92,100	73,700	52.8	41.1	11.8	72,900	52.8	40.4	12.5	− 0.7
Ireland	70,200	63,300	75.2	72.5	2.7	67,800	75.2	74.3	0.9	1.8
Italy	290,000	571,000	24.3	61.5	− 37.2	566,000	24.3	61.2	− 36.9	− 0.3
Lithuania	64,100	57,500	57.0	52.0	5.0	52,700	57.0	47.7	9.3	− 4.3
Luxembourg	2500	26,800	75.6	97.7	− 22.1	26,600	75.6	97.7	− 22.1	0.0
Latvia	64,200	43,300	79.5	69.6	9.9	40,300	79.5	67.3	12.2	− 2.3
Malta	238	3800	26.0	95.4	− 69.4	5300	26.0	96.7	− 70.7	1.3
Netherlands	35,700	336,000	69.9	96.8	− 26.9	371,000	69.9	97.1	− 27.2	0.3
Poland	310,000	310,000	36.8	36.8	0.0	307,000	36.8	36.1	0.7	− 0.7
Portugal	88,700	183,000	37.5	69.7	− 32.2	166,000	37.5	66.6	− 29.2	− 3.0
Romania	236,000	183,000	36.2	17.7	18.5	186,000	36.2	18.9	17.3	1.2
Sweden	394,000	424,000	38.5	42.9	− 4.5	431,000	38.5	43.8	− 5.4	0.9
Slovenia	20,300	24,400	49.7	58.0	− 8.3	23,100	49.7	55.7	− 6.0	− 2.3
Slovakia	48,900	37,600	69.4	60.2	9.2	38,700	69.4	61.4	8.0	1.2
United Kingdom	248,000	515,000	22.0	62.3	− 40.4	581,000	22.0	66.6	− 44.6	4.3
United States	7,740,000	7,840,000	23.9	24.8	− 1.0	8,030,000	23.9	26.6	− 2.7	1.8
Japan	410,000	1,220,000	5.0	68.2	− 63.1	1,360,000	5.0	71.5	− 66.4	3.3
China	6,990,000	12,300,000	15.8	51.9	− 36.2	12,100,000	15.8	51.4	− 35.6	− 0.6
Canada	3,410,000	2,700,000	29.7	11.0	18.7	2,710,000	29.7	11.4	18.3	0.5
South Korea	105,000	719,000	11.7	87.1	− 75.4	754,000	11.7	87.7	− 76.1	0.6
Brazil	6,950,000	5,810,000	19.4	3.5	15.9	5,750,000	19.4	2.5	16.9	− 1.0
India	3,070,000	3,390,000	9.4	18.1	− 8.7	3,430,000	9.4	19.1	− 9.7	1.0
Mexico	1,910,000	1,710,000	26.2	17.5	8.7	1,710,000	26.2	17.9	8.3	0.4
Russia	10,200,000	7,110,000	33.6	4.9	28.7	7,000,000	33.6	3.5	30.1	− 1.5
Australia	4,870,000	1,800,000	64.9	5.1	59.8	1,860,000	64.9	8.3	56.6	3.2
Switzerland	36,000	72,600	49.4	74.9	− 25.5	79,200	49.4	77.0	− 27.6	2.1
Turkey	761,000	971,000	13.1	31.9	− 18.8	959,000	13.1	31.0	− 18.0	− 0.8
Taiwan	35,800	1,230,000	48.6	98.5	− 49.9	990,000	48.6	98.1	− 49.5	− 0.4
Norway	262,000	209,000	47.6	34.3	13.3	207,000	47.6	33.6	14.0	− 0.7
Indonesia	1,810,000	2,160,000	16.1	29.6	− 13.6	2,200,000	16.1	31.1	− 15.0	1.5
South Africa	1,190,000	952,000	28.7	10.5	18.2	935,000	28.7	8.9	19.8	− 1.6
RoW Asia and Pacific	8,810,000	8,820,000	21.8	21.9	0.0	8,460,000	21.8	18.6	3.2	− 3.3
RoW America	8,120,000	7,240,000	24.0	14.8	9.2	7,470,000	15.7	8.4	7.2	1.9
RoW Europe	1,090,000	711,000	46.3	17.8	28.5	770,000	37.7	11.9	25.7	2.8
RoW Africa	17,200,000	14,800,000	19.4	6.7	12.7	14,900,000	16.6	3.6	13.0	− 0.3
RoW Middle East	1,110,000	2,280,000	26.6	64.4	− 37.8	2,280,000	15.0	58.7	− 43.7	5.9
Total	90,300,000	90,300,000	25.8	25.8	0.0	90,300,000	24.2	24.2	0.0	0.0

BLET is the export share out of total consumption minus the import share out of total consumption. BLET difference is the percentage difference in BLET between the databases

The top 20 products (global aggregation of results across all countries) ranked according to aggregation error of land embodied in imports are displayed in Table 3. Remembering that the impacts embodied in imports originating in the non-RoW regions are identical in the aggregated and disaggregated database, these results reflect the effect of disaggregation purely of the EXIOBASE 3 RoW regions. The land embodied in imports associated with “Products of forestry, logging and related services (02)” is the single largest product group, with 66,10,000 km^2^ or 30.2% of total global land use embodied in imports. This product group is somewhat susceptible to regional aggregation error, with a summed difference between the aggregated and disaggregated database of 6,60,000 km^2^ or 19.4% of the total aggregation error observed between the models. In contrast, for “Meat animals nec” and “Hotel and restaurant services (05) “the share of land use embodied in exports is only in the range of 1–2%, but the aggregation error of the product relative to the flow (shown by the “error score”) is much higher at 64% and 95% of the value of the estimated flow, respectively. This suggests a large degree of uncertainty due to regional aggregation in the aggregated database. The last column of Table 3 shows that the aggregation can change the value of the flow by a factor of over five (“Copper ores and Concentrates”) where the value in the aggregated database is 17% of the corresponding value in EXIOBASE 3rx.

**Table 3:** Top 20 product aggregation error of land embodied in imports (2015)

Product	Total land area of flow (km^2^)	Share of global land area (km^2^), %	Aggregation error (km^2^)	Error score (*ε*)	Share of total aggregation error, %	Difference between databases (100% is equal to no difference), %
Products of forestry, logging and related services (02)	6,610,000	30.2	660,000	0.10	19.4	95
Oil seeds	1,770,000	8.1	251,000	0.14	7.4	95
Hotel and restaurant services (55)	223,000	1.0	209,000	0.93	6.1	159
Meat animals nec	327,000	1.5	208,000	0.64	6.1	126
Wood and products of wood and cork (except furniture); articles of straw and plaiting materials (20)	1,150,000	5.3	135,000	0.12	4.0	92
Products of meat cattle	1,810,000	8.3	127,000	0.07	3.7	98
Food products nec	762,000	3.5	118,000	0.15	3.5	108
Chemicals nec	406,000	1.9	117,000	0.29	3.4	96
Vegetables, fruit, nuts	643,000	2.9	104,000	0.16	3.1	106
Wheat	1,020,000	4.7	104,000	0.10	3.0	91
Copper ores and concentrates	98,800	0.5	93,300	0.94	2.7	17
Cereal grains nec	755,000	3.5	78,400	0.10	2.3	93
Other business services (74)	55,100	0.3	52,500	0.95	1.5	151
Crops nec	374,000	1.7	51,200	0.14	1.5	98
Real estate services (70)	38,200	0.2	50,500	1.32	1.5	181
Cattle	1,510,000	6.9	48,800	0.03	1.4	101
Crude petroleum and services related to crude oil extraction, excluding surveying	103,000	0.5	44,300	0.43	1.3	94
Dairy products	529,000	2.4	40,600	0.08	1.2	99
Furniture; other manufactured goods n.e.c. (36)	233,000	1.1	39,600	0.17	1.2	113
Construction work (45)	53,600	0.2	39,100	0.73	1.1	147

Ranked according to percentage of total product aggregation error. The error score is relative to the total value of the specific flow of imports. The share of total aggregation error refers to the aggregation error summed across all flows (i.e., global). The difference between databases shows the value of the flow in the aggregated database compared to that in EXIOBASE 3rx

The aggregation error for land embodied in imports for regions sorted by regional error score (Table 4) shows that the countries with the largest scores, such as Australia and Malta, have a low share of global imports, although the net effect of the aggregation error for the countries is significant. Countries with a low import share out of total consumption of land, such as Russia, Brazil and Australia (Table 2) have the largest aggregation errors. In addition, these countries stand out with a high proportion of land originating in EXIOBASE 3 RoW regions. A large share of the regional aggregation error is centered in Asia due to Taiwan and Japan having relatively larger aggregation error shares than land import shares, combined with China dominating land imports (although the aggregation error is relatively lower).

**Table 4:** Land embodied in imports and aggregation error of 49 regions (2015)

Region	Total land area of flow (km^2^)	Share of global land area (km^2^), %	Aggregation error (km^2^)	Error score (*ε*)	Share of total aggregation error, %	Difference between databases (100 is equal to no difference), %
AU	92,300	0.4	67,400	0.73	2.0	168
MT	3620	0.0	2050	0.57	0.1	141
BR	203,000	0.9	83,100	0.41	2.4	70
RU	350,000	1.6	143,000	0.41	4.2	69
FR	442,000	2.0	161,000	0.37	4.7	95
ZA	99,900	0.5	35,600	0.36	1.0	84
CH	54,400	0.2	18,000	0.33	0.5	112
GB	321,000	1.5	100,000	0.31	2.9	121
HR	10,000	0.0	3010	0.30	0.1	107
IN	614,000	2.8	183,000	0.30	5.4	107
ES	217,000	1.0	63,400	0.29	1.9	113
RO	32,300	0.1	9200	0.28	0.3	108
PT	127,000	0.6	34,900	0.27	1.0	87
LU	26,200	0.1	7030	0.27	0.2	99
BE	217,000	1.0	57,600	0.27	1.7	87
SI	14,200	0.1	3740	0.26	0.1	91
GR	48,600	0.2	12,700	0.26	0.4	99
TW	1,210,000	5.6	315,000	0.26	9.2	80
NO	71,700	0.3	16,900	0.24	0.5	97
TR	310,000	1.4	72,500	0.23	2.1	96
DK	53,200	0.2	12,200	0.23	0.4	102
LT	29,900	0.1	6730	0.23	0.2	84
NL	325,000	1.5	71,600	0.22	2.1	111
IT	351,000	1.6	75,200	0.21	2.2	99
DE	573,000	2.6	112,000	0.19	3.3	103
JP	834,000	3.8	160,000	0.19	4.7	117
IE	45,900	0.2	7890	0.17	0.2	110
HU	30,300	0.1	4930	0.16	0.1	97
WM	1,350,000	6.2	213,000	0.16	6.2	99
BG	14,600	0.1	2210	0.15	0.1	108
PL	114,000	0.5	16,800	0.15	0.5	97
WE	100,000	0.5	14,600	0.15	0.4	92
CY	4460	0.0	636	0.14	0.0	106
AT	78,000	0.4	10,900	0.14	0.3	101
US	1,950,000	8.9	252,000	0.13	7.4	110
KR	626,000	2.9	76,700	0.12	2.3	106
LV	30,200	0.1	3530	0.12	0.1	90
ID	639,000	2.9	73,800	0.12	2.2	107
CZ	48,000	0.2	5400	0.11	0.2	102
CN	6,360,000	29.1	677,000	0.11	19.8	98
EE	11,500	0.1	1180	0.10	0.0	98
SK	22,600	0.1	2280	0.10	0.1	105
SE	182,000	0.8	16,400	0.09	0.5	104
WF	530,000	2.4	46,700	0.09	1.4	100
CA	296,000	1.4	23,700	0.08	0.7	105
WA	1,580,000	7.3	100,000	0.06	2.9	100
MX	298,000	1.4	14,200	0.05	0.4	103
FI	284,000	1.3	5690	0.02	0.2	100
WL	622,000	2.8	12,200	0.02	0.4	101

Sorted by aggregation error score. The error score is relative to the total value of the specific flow of imports. The share of total aggregation error refers to the aggregation error summed across all flows (i.e., global). The difference between databases shows the value of the flow in the aggregated database compared to that in EXIOBASE 3rx

Digging deeper into the land embodied in imports by also showing the traded product (Additional file 1: Table S1), we find that the six largest product- and region-specific aggregation errors are due to imports for Taiwan, China and India. Together, they make up about 19% of global aggregation error of land embodied in imports. Asian countries dominate the top 20 list. We also notice that certain items, such as imports of “Hotel and restaurant services (55)” to China and “Meat animals nec” to Japan have significant aggregation error scores. The net effect of the aggregation can change results by up to an order of magnitude (“Chinese imports of Hotel and restaurant services (55)”).

By also including the origin region of the imported good, the concentration of the aggregation error around Asian regions and “Products of forestry, logging and related services (02)” becomes even more apparent (Additional file 1: S8). The total global aggregation error is concentrated on a few flows, with the top 20 contributors to the error summing up to 25% of the global total error. 12 of the top 20 flows are imports originating in RoW Asia.

## Discussion

### Hotspots for aggregation errors of land embodied in trade

Countries such as China show sharp trends of rapid increases in imports in the later years, and as such also become the main importers of traded land (see Additional file 3). Results show that there is a need for the integration and calculation of a high level of regional detail in these countries’ trade partners to avoid regional aggregation errors. We find that the import aggregation errors of Asian countries such as China, India, Taiwan and Japan make up a large share of the global total error (Table 4). Although RoW Asia contributes to only 7.2% of global exported land, the contribution to the export aggregation error is 47.9% (Additional file 1: S8).

The effect of regional aggregation on land embodied in trade by products shows a large concentration of both land embodied in trade and aggregation errors around a handful of products (Table 3). The products are mostly part of the forestry and agricultural sectors, with a few outliers in the service sectors such as “Hotels and restaurant services (55)”, “Other business services (74)” and “Real estate services (70)”. These outliers are characterized by low shares of total land embodied in trade, but relatively larger shares of aggregation errors. The same is the case for some of the more disparate products groups (those in the not elsewhere classified groups). These later results indicate the need for also more detailed sectoral resolution (see below).

The regions and products prone to aggregation errors depend on the year chosen. We chose to present results for 2015 in this paper, as this is the most recently available data in EXIOBASE 3rx. A look into the aggregation errors summed together across the whole time series (Additional file 1: S8, and S9 for 2015) reveals that 37.4% of the export aggregation error now comes from RoW Africa (27.1% in 2015), while RoW Asia is responsible for 45.6% of the global total (47.9% in 2015). The import aggregation errors for regions show the same trends, except for Portugal that now ranks third when sorting by regions. The products most heavily affected by the aggregation throughout the time series show similar trends to the equivalent 2015 result, but even more concentrated around products of forestry, logging and related services (02) which accounts for 25.9% of the total aggregation error across the full time series (19.4% in 2015). Including the origin and destination of imports reveals that the top four flows, making up 12% of the total aggregation error, are “Products of forestry, logging and related services (02)” from RoW Africa to China, Portugal, India and France.

Compared to other works, Kastner et al. (2014) found that MRIO studies on cropland embodied in Chinese trade diverged from studies using other methods. We find that China’s balance of land embodied in trade for all land types (Table 2) did not significantly differ between the two levels of regional aggregation. Despite not finding an aggregation effect, we find a significant change in China’s balance of cropland embodied in trade from 1995 to 2015 (Additional file 1: S6). From 1995 to 2000 China was a net exporter of cropland, while from 2001 to 2015 there is a shift to becoming a net importer and increasingly so as we approach present time. Although our results use monetary values for the trade allocation, while studies using other methods typically use physical properties, the time trend we find should be interesting for future research looking at the deviations in results between methods.

Given that a few countries import a large share of globally traded land, we find it is particularly important to have their trading partners represented as individual regions in MRIOs. Similarly, key exporting regions not currently included, such as Argentina, should be represented, and large countries (such as China) can even be split into sub-regions as suggested by Su and Ang (2010) to minimize aggregation errors.

### Challenges and limitations

The inclusion of 214 countries in a single database comes with a trade-off in terms of raw data availability and uncertainty. Whilst country-specific land use, production, and trade data are used (for an overview of the regional data availability in the raw data, see Additional file 1: S2), a lot of data estimation is undertaken, especially for the countries not originally in the EXIOBASE dataset. For the 44 countries originally in the EXIOBASE 3 dataset, it would be expected that the additional disaggregation of the rest-of-the-world regions would improve accuracy. However, for the remaining countries, it must be expected that the uncertainty of individual country estimates are high. Especially when disaggregating small (and trade-exposed) countries the expectation of accuracy is low. It is common in all input–output studies (and all statistical data) to find a declining relationship between accuracy and volume (whether expressed as GDP, output, or key coefficients) (see for example (Lenzen et al. 2010, Karstensen et al. 2015, Wood et al. 2019)) for one reason because of the laws of error propagation (Imbeault-Tétreault et al. 2013). Whilst further work could see the replacement of generic data with more country-specific data, it is still likely that the uncertainty levels of individual countries in the disaggregated database will be high, and it is anticipated that the further development of single-country national account consistent procedures are further developed in order to undertake county specific analysis (see, e.g., Edens et al. (2015); Palm et al. (2019); Hambÿe et al. (2018)).

In terms of empirical validation of results as presented, there are sudden jumps in per capita land footprint results, particularly for small economies such as Aruba, San Marino, Bermuda, the Cayman Islands and the British Virgin Islands (as can be seen in Additional file 1: S6). In addition to being small economies, several of these countries heavily rely on imports with import shares in the range of 95–98% of the total consumption-based land footprints, except for the British Virgin Islands and the Cayman Islands where this value is 43.0% and 50.7%, respectively (see Additional file 1: S13). When there is a jump in land footprint, we do note that that there are sudden changes in the import structure for the specific years (see https://oec.world/en/ (Simoes and Hidalgo 2011) for a visualization of trade data). Aruba has a drastic increase in imports of cattle from Sudan (2010), Bermuda and the British Virgin Islands import crude petroleum from Kazakhstan (2000–2003), San Marino imports raw fur skins from Russia (2006), while the Cayman Islands import soybeans from Paraguay (2001–2007). Drastic increases in imports of these specific products from countries with high use of land area per monetary output, combined with high import shares drastically change the per capita footprint of these countries using the EEBT approach. The EEBT approach however, does not allow us to determine whether these imports are used for domestic consumption, or intermediate production that is later used for exports and therefore should not be counted in that country’s consumption-based footprints.

In terms of data reconciliation issues, most of the challenges in building EXIOBASE 3rx were related to the SUT balancing where there were contradictions between the initial estimates and the macroeconomic data. Several of these issues were resolved by changing options in the balancing routine that increased the accepted level of deviation (which was set to a cap in the balancing) from the initial estimated SUTs. If this did not work, we used initial technical coefficient estimates from EXIOBASE 2. In several of the remaining unbalanced cases (Additional file 1: S5), the issue is negative value added from the macroeconomic data specifically for International Standard Industrial Classification C and E from the UN National Main Aggregates Database. Resolving this issue is a work in progress. There are a total of 151 cases with a non-optimal solution in the SUT balancing over the time series (3 cases for year 2015). Data for these cases are set to zero and sum up to 0.15% of global GDP through the time series, hence it should not significantly influence the overall results. To resolve the balancing issues would require more detailed and reliable raw data, which again would manifest in the balancing routine deviating less from the initial estimated SUTs.

Setting the unbalanced countries to zero lead to a slight imbalance in land footprint results (see Table 2). This is one of several ways of dealing with such imbalances. In Eora, this has been handled by compiling the unbalanced regions in a Rest-of-the-world region (Lenzen et al. 2013). As setting the values of the environmental extensions matrix (**F**) to zero for an unbalanced country A means neglecting the land use embodied in imports of a country B from country A, there is a slight underreporting of land use in EXIOBASE 3rx. In 2015, Puerto Rico and the Dominican Republic are the countries whose total land footprints are affected the most by this, with an underreported footprint of 0.86%. For the aggregated database this effect has different distributional impacts as it affects all countries that import from the RoW region that country A is aggregated to. In addition, it affects the domestic part of the RoW region’s footprint as there is not a one-to-one relationship between the output of country A and the land use per unit of output (**S**). RoW America’s land footprint is affected heaviest by this with a change of 0.25%. In the 49-region version of EXIOBASE 3rx, the change is largest in Latvia (0.08%). Resolving the issue with unbalanced regions in EXIOBASE 3rx is a work in progress.

Using the EEBT approach, we do not distinguish between intermediate and final use of traded products. The approach fits with the scope of this paper as we look at the land embodied in aggregated imports and exports. The EEBT approach is also argued to be more relevant for global trade-related policy (Peters 2007). However, when allocating impacts to categories of final demand, the EEBT approach will give different results compared to the Leontief approach due to different allocation of impacts, although the global total impact is the same. For a country, imported goods that are used for intermediate production, and later exported are in the EEBT approach accounted as part of the imported footprint, while in the MRIO approach, they are not. The implications of this are discussed in Peters (2007). The extent of the difference between the two approaches is unexplored in this paper, although previous studies indicate that this difference could be significant (Su and Ang 2011).

In terms of land use data, other types of area use such as ocean are sometimes included in land use studies (e.g., Weinzettel et al. (2013)). This could alter the regional results, the land embodied in trade, and most likely the hotspots for large aggregation errors, through, e.g. consumption of fish (Weinzettel et al. 2013). It is important to be aware that the effects due to regional aggregation are sensitive to the types of land included in the study. Similarly, the picture would likely look different in terms of regions and sectors sensitive to aggregation errors when studying other types of environmental impacts. For example de Koning et al. (2015) found that regional aggregation had small effects on overall carbon and material footprints. Bouwmeester and Oosterhaven (2013) on the other hand find large, and what they refer to as unacceptable aggregation errors for particularly water use, but also for CO_2_ emissions, although their regional aggregation is more drastic with aggregating 43 regions to five and two regions. The deviating conclusions on the effect of regional aggregation in other papers suggest that there is still need for further research on both the underlying causes of differences in these results, as well as identifying regions that are sensitive to aggregation errors. Although de Koning et al. (2015) look at different indicators, our findings coincide in the sense that when looking at the footprint of a country, the net effect of a regional aggregation is not drastic, but when exploring products traded and trade partners in more detail we find large effects of aggregation. This could also manifest in larger deviations when aggregating to very few regions, as in Bouwmeester and Oosterhaven (2013).

### Further work

The results at hand are the first published results using EXIOBASE 3rx. We restrict our scope to the effect of regional aggregation of land use embodied in trade. However, with the limitations related to the EEBT approach and unbalanced countries in mind, there is still unexplored potential in using the database for land use studies in its current form. Firstly, there are multiple land use extensions available, which allows for studying different land types embodied in trade. Secondly, land use embodied in trade can be studied on a sectoral level as the database includes 200 products harmonized across all regions. Thirdly, the database is a time series from 1995 to 2015 which allows for studying the drivers of land use in form of panel regressions or similar methods. This creates opportunities for following up literature findings that suggest some degree of correlation between income and land use (Weinzettel et al. 2013; Ivanova et al. 2016). Panel regression studies using MRIO time series data also enable predictions into the future, which could help overcome the retrospective scope that is identified as a limitation of MRIO studies, which again could increase policy relevance (Axtell et al. 2001).

Currently only land extensions are processed for EXIOBASE 3rx. However, adding other environmental extensions to the database is a work in progress. More immediately, we chose land use as it is a simple and key indicator of agricultural related impacts. The application of biodiversity characterization factors (Verones et al. 2017) and net-primary productivity (Kastner et al. 2015; Weinzettel et al. 2019) are simple extensions to obtain more policy-relevant work. Furthermore, the correlation (Silva Simas et al. 2017) of land use with other agricultural impacts such as blue water consumption (Lutter et al. 2016) and eutrophication (Hamilton et al. 2018) gives a good basis for further extension.

Regarding resolution, the sectoral resolution in EXIOBASE is one of the most detailed in the available MRIOs (Steen-Olsen et al. 2014). However, despite the comparably high sectoral resolution of EXIOBASE 3rx, the sectoral resolution is a main point of criticism and source of error of land use studies using MRIO (Bruckner et al. 2015; Weinzettel et al. 2013; Steen-Olsen et al. 2012). Disaggregation of sectors is argued by Weinzettel et al. (2014) to be an important future development of MRIOs, and can replace the hybrid approaches applied to overcome this limitation today. Already we are seeing the linking of detailed FAO production and use data to both aggregated and disaggregated MRIO tables (Weinzettel et al. 2019) and even the construction of country-specific physical input–output tables (Bruckner et al. 2019).

In terms of methods, there is further work on expanding the cross-entropy model (Többen and Schröder 2018) used for reconciling the bilateral trade data with main aggregates of national accounts and estimates of product output, first, to the balancing of the SUTs and, later, to the simultaneous reconciliation of bilateral trade, SUTs and the physical extensions. The main challenges for the practical implementation of such a concept are the computational requirements due to the enormous size of the database (see the method section for a brief overview of the size of EXIOBASE 3rx). However, recent theoretical work on topological transformations (Rodrigues et al. 2016) and maximum entropy models to reconcile data in physical and monetary units simultaneously (Többen 2017) constitute first theoretical steps to solve this issue.

## Conclusion

With divergence in environmental results between MRIOs hampering the policy relevance of MRIO studies, it is important to both develop more detailed models, and to get a systematic understanding about the underlying sources of these differences. We have developed a regional extension of EXIOBASE 3 called EXIOBASE 3rx and studied the effect of regional aggregation on land use embodied in trade by comparing results to an aggregated version of the same database consisting of 49 regions. Whilst the disaggregated database is experimental in that a lot of structural economic data are estimated, country-specific data on agricultural and resource output, as well as trade are included. We find that the regional aggregation error for land use embodied in imports on a sectoral level is highly concentrated on sectors with high biomass demand, such as forestry, meat from animals, wood products and hotels and restaurant services. The effect on regions shows that the balance of land embodied in trade differs with up to 6% between the aggregate database and EXIOBASE 3rx, while the net aggregation error of land embodied in imports for some of the 49 EXIOBASE regions differ up to 68% between the databases. The largest absolute aggregation errors for land embodied in imports are found for Asian imports particularly originating in RoW Asia and RoW Africa.

Our findings have two important implications regarding the use of MRIOs for land use studies. Firstly, regions in Asia and Africa should be represented in detail, and higher sectoral disaggregation is necessary for a handful of key sectors. Secondly, we suggest that MRIO developers are aware of the potentially significant effects of regional aggregation and build MRIOs that find the right balance between number of regions and sectors for their studies, while at the same time acknowledging the potential uncertainty introduced by assumptions aimed at closing data gaps in raw data. Further research is needed to identify key sectors and regions vulnerable to aggregation errors. If these are found to converge across environmental and socioeconomic extensions, MRIO systems can be built that find the right level of detail without becoming unnecessarily large. We believe that this is an important step in finding the sources of intra-MRIO result discrepancies and could increase the policy uptake of MRIO studies.

## Supplementary information

**Additional file 1.** Supplementary material.

**Additional file 2.** Concordance matrices for regions, land use types and industries.

**Additional file 3.** Land use time series results (1995–2015).

## Data Availability

The database generated and analyzed during the current study are available in the EXIOBASE 3rx repository, https://zenodo.org/deposit/2654460. Most of the raw data used to build EXIOBASE 3rx, as referred to in the manuscript, is publically available at the repositories listed in the list of references. The land use dataset is available in an aggregate format in the EXIOBASE 3rx repository. The full dataset is available from the corresponding authors (MT, TK and KHE) on reasonable request.

## Abbreviations

EEBT: Emissions embodied in bilateral trade
MRIO: Multiregional input output
RoW: Rest of the world
SUT: Supply–use table
