# Supplementary material for: Adding country resolution to EXIOBASE: impacts on land use embodied in trade
Source: J Econ Struct. 2020 Feb 13;9(1):14. doi: 10.1186/s40008-020-0182-y (PMC7021151; doi:10.1186/s40008-020-0182-y)
Supplement: Supplementary file 1 — Additional file 1. Supplementary material. [file 40008_2020_182_MOESM1_ESM.docx]

Supporting Information

# Workflow differences: EXIOBASE 3 vs EXIOBASE 3rx

The workflows of EXIOBASE 3rx and EXIOBASE 3 (from Stadler et al. (2018)) are represented in Figure S 1 and Figure S 2 respectively. Here we list the differences between the workflows with reference to the relevant parts of the workflow in Figure S 1 in parentheses:

- The trade balancing routine is updated to a maximum entropy approach (Part A)
- Initial coefficients from time series of EXIOBASE 3 or EXIOBASE 2 (Part C)
  - 5 RoW regions coefficients used for 170 countries
- All raw data available processed on an individual country level instead of aggregated to RoW regions as in previous versions of EXIOBASE (Part A and B)
- Balanced macro-economic time series data for 214 countries instead of 49 regions
- SUT balanced for basic price only
  - No taxes or trade and transport margins estimated as individual layers
- UN service trade data reconciled simultaneously with trade balancing, not separately.
- If no raw data was available for product output estimates, we used the shares in EXIOBASE 3 to disaggregate the UN estimates of gross output
- Minor changes to product/sector bridging between EXIOBASE sectors and e.g. FAOSTAT, IEA and UN service trade data.
- Minor changes to gap filling pre-SUT balancing


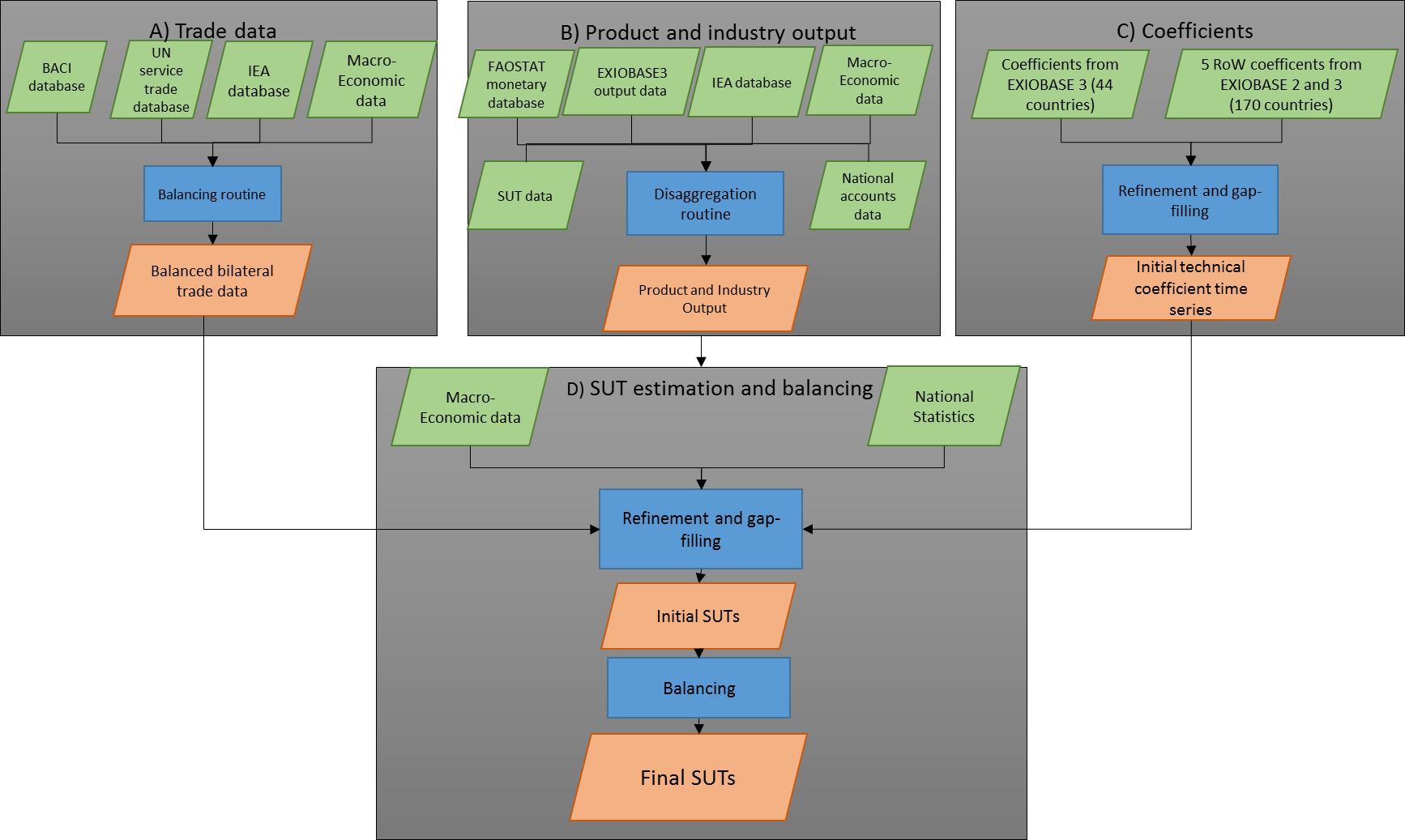


Figure S 1: Workflow EXIOBASE 3rx


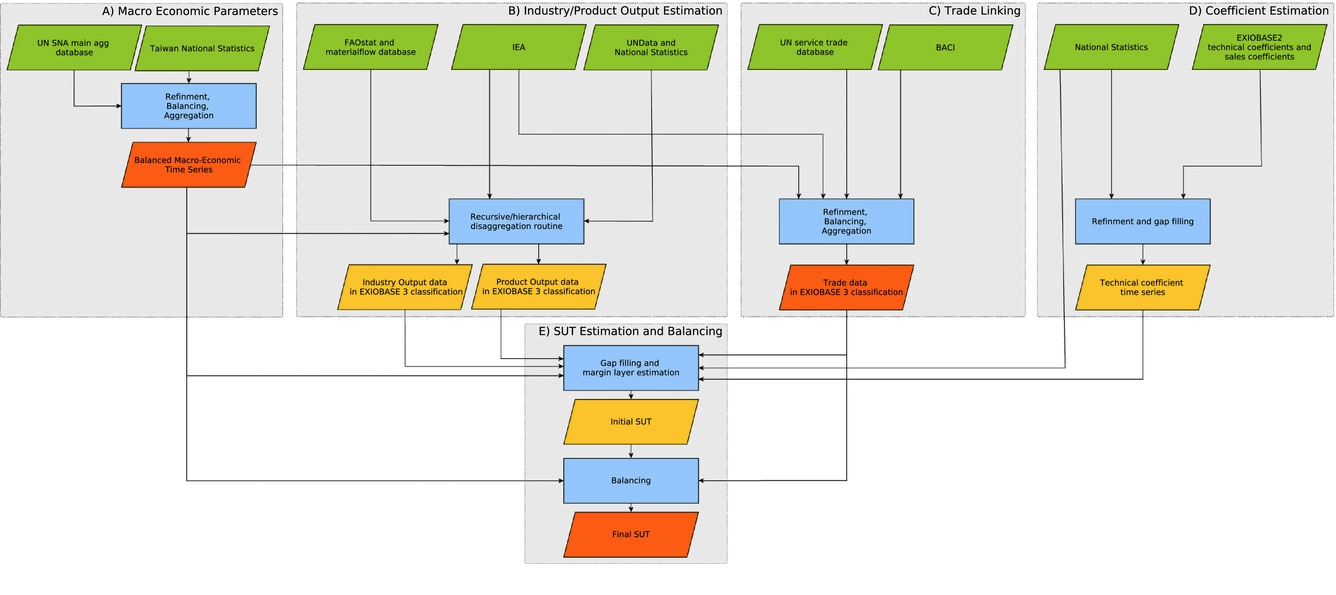


Figure S 2: EXIOBASE 3 workflow from Stadler et al. (2018)

# Data sources

| **Codes** |
| --- |
| 1 - Real |
| 2 – Splitting RoW regions based on GDP |
| 3 – Proxy country used |
| 4 –Rest-of-the-world coefficients |

Table S 1: Data sources EXIOBASE 3rx

|  | **Macro-economic data from EXIOBASE 3^[[1]](#footnote-1)^** **^[[2]](#footnote-2)^** | **Trade^[[3]](#footnote-3)^ (BACI)** | **IEA^[[4]](#footnote-4)^** | **FAO data^[[5]](#footnote-5)^** | **Material data from EXIOBASE 3**2 | **UN Service trade data from EXIOBASE 3**2 | **SUT coefficients from EXIOBASE 3 (and 2)** | **Land use data (see S11)** |
| --- | --- | --- | --- | --- | --- | --- | --- | --- |
| Afghanistan | 1 | 1 | 2 | 1 | 2 | 2 | 4 | 1 |
| Albania | 1 | 1 | 1 | 1 | 2 | 2 | 4 | 1 |
| Algeria | 1 | 1 | 1 | 1 | 2 | 2 | 4 | 1 |
| Andorra | 1 | 1 | 3 | 3 | 2 | 2 | 4 | 1 |
| Angola | 1 | 1 | 1 | 1 | 2 | 2 | 4 | 1 |
| Anguilla | 1 | 1 | 2 | 3 | 2 | 2 | 4 | 1 |
| Antigua and Barbuda | 1 | 1 | 2 | 1 | 2 | 2 | 4 | 1 |
| Argentina | 1 | 1 | 1 | 1 | 2 | 2 | 4 | 1 |
| Armenia | 1 | 1 | 1 | 1 | 2 | 2 | 4 | 1 |
| Aruba | 1 | 1 | 2 | 3 | 2 | 2 | 4 | 1 |
| Australia | 1 | 1 | 1 | 1 | 1 | 1 | 1 | 1 |
| Austria | 1 | 1 | 1 | 1 | 1 | 1 | 1 | 1 |
| Azerbaijan | 1 | 1 | 1 | 1 | 2 | 2 | 4 | 1 |
| Bahamas | 1 | 1 | 2 | 1 | 2 | 2 | 4 | 1 |
| Bahrain | 1 | 1 | 1 | 1 | 2 | 2 | 4 | 1 |
| Bangladesh | 1 | 1 | 1 | 1 | 2 | 2 | 4 | 1 |
| Barbados | 1 | 1 | 2 | 1 | 2 | 2 | 4 | 1 |
| Belarus | 1 | 1 | 1 | 1 | 2 | 2 | 4 | 1 |
| Belgium | 1 | 2 | 1 | 1 | 1 | 1 | 1 | 1 |
| Belize | 1 | 1 | 2 | 1 | 2 | 2 | 4 | 1 |
| Benin | 1 | 1 | 1 | 1 | 2 | 2 | 4 | 1 |
| Bermuda | 1 | 1 | 2 | 1 | 3 | 3 | 4 | 1 |
| Bhutan | 1 | 1 | 2 | 1 | 2 | 2 | 4 | 1 |
| Bolivia | 1 | 1 | 1 | 1 | 2 | 2 | 4 | 1 |
| Bosnia and Herzegovina | 1 | 1 | 1 | 1 | 2 | 2 | 4 | 1 |
| Botswana | 1 | 2 | 1 | 1 | 2 | 2 | 4 | 1 |
| Brazil | 1 | 1 | 1 | 1 | 1 | 1 | 1 | 1 |
| British Virgin Islands | 1 | 1 | 2 | 1 | 2 | 2 | 4 | 3 |
| Brunei Darussalam | 1 | 1 | 1 | 1 | 2 | 2 | 4 | 1 |
| Bulgaria | 1 | 1 | 1 | 1 | 2 | 2 | 1 | 1 |
| Burkina Faso | 1 | 1 | 2 | 1 | 2 | 2 | 4 | 1 |
| Burundi | 1 | 1 | 2 | 1 | 2 | 2 | 4 | 1 |
| Cabo Verde | 1 | 1 | 2 | 1 | 2 | 2 | 4 | 1 |
| Cambodia | 1 | 1 | 1 | 1 | 2 | 2 | 4 | 1 |
| Cameroon | 1 | 1 | 1 | 1 | 2 | 2 | 4 | 1 |
| Canada | 1 | 1 | 1 | 1 | 1 | 1 | 4 | 1 |
| Cayman Islands | 1 | 1 | 2 | 1 | 2 | 2 | 4 | 3 |
| Central African Republic | 1 | 1 | 2 | 1 | 2 | 2 | 4 | 1 |
| Chad | 1 | 1 | 2 | 1 | 2 | 2 | 4 | 1 |
| Chile | 1 | 1 | 1 | 1 | 2 | 2 | 4 | 1 |
| China | 1 | 1 | 1 | 1 | 1 | 1 | 1 | 1 |
| Hong Kong | 1 | 1 | 1 | 1 | 2 | 2 | 4 | 1 |
| Macao | 1 | 1 | 2 | 1 | 3 | 3 | 4 | 3 |
| Colombia | 1 | 1 | 1 | 1 | 2 | 2 | 4 | 1 |
| Comoros | 1 | 1 | 2 | 1 | 2 | 2 | 4 | 1 |
| Congo Republic | 1 | 1 | 1 | 1 | 2 | 2 | 4 | 1 |
| Cook Islands | 1 | 1 | 2 | 1 | 2 | 2 | 4 | 1 |
| Costa Rica | 1 | 1 | 1 | 1 | 2 | 2 | 4 | 1 |
| Croatia | 1 | 1 | 1 | 1 | 1 | 1 | 4 | 1 |
| Cuba | 1 | 1 | 1 | 1 | 2 | 2 | 4 | 1 |
| Curacao | 1 | 1 | 2 | 3 | 2 | 2 | 4 | 3 |
| Cyprus | 1 | 1 | 1 | 1 | 1 | 1 | 4 | 1 |
| Czech Republic | 1 | 1 | 1 | 1 | 1 | 1 | 1 | 1 |
| Cote d'Ivoire | 1 | 1 | 1 | 1 | 2 | 2 | 4 | 1 |
| North Korea | 1 | 1 | 1 | 1 | 3 | 3 | 4 | 1 |
| DR Congo | 1 | 1 | 1 | 1 | 2 | 2 | 4 | 1 |
| Denmark | 1 | 1 | 1 | 1 | 1 | 1 | 1 | 1 |
| Djibouti | 1 | 1 | 2 | 1 | 2 | 2 | 4 | 1 |
| Dominica | 1 | 1 | 2 | 1 | 2 | 2 | 4 | 1 |
| Dominican Republic | 1 | 1 | 1 | 1 | 2 | 2 | 4 | 1 |
| Ecuador | 1 | 1 | 1 | 1 | 2 | 2 | 4 | 1 |
| Egypt | 1 | 1 | 1 | 1 | 2 | 2 | 4 | 1 |
| El Salvador | 1 | 1 | 1 | 1 | 2 | 2 | 4 | 1 |
| Equatorial Guinea | 1 | 1 | 2 | 1 | 2 | 2 | 4 | 1 |
| Eritrea | 1 | 1 | 1 | 1 | 2 | 2 | 4 | 1 |
| Estonia | 1 | 1 | 1 | 1 | 1 | 1 | 1 | 1 |
| Ethiopia | 1 | 1 | 1 | 1 | 2 | 2 | 4 | 1 |
| Fiji | 1 | 1 | 2 | 1 | 3 | 3 | 4 | 1 |
| Finland | 1 | 1 | 1 | 1 | 1 | 1 | 1 | 1 |
| Netherlands Antilles | 1 | 1 | 1 | 1 | 3 | 3 | 4 | 1 |
| France | 1 | 1 | 1 | 1 | 1 | 1 | 1 | 1 |
| French Polynesia | 1 | 1 | 2 | 1 | 3 | 3 | 4 | 1 |
| Gabon | 1 | 1 | 1 | 1 | 2 | 2 | 4 | 1 |
| Gambia | 1 | 1 | 2 | 1 | 2 | 2 | 4 | 1 |
| Georgia | 1 | 1 | 1 | 1 | 2 | 2 | 4 | 1 |
| Germany | 1 | 1 | 1 | 1 | 1 | 1 | 1 | 1 |
| Ghana | 1 | 1 | 1 | 1 | 2 | 2 | 4 | 1 |
| Greece | 1 | 1 | 1 | 1 | 1 | 1 | 1 | 1 |
| Greenland | 1 | 1 | 2 | 1 | 2 | 2 | 4 | 1 |
| Grenada | 1 | 1 | 2 | 1 | 2 | 2 | 4 | 1 |
| Guatemala | 1 | 1 | 1 | 1 | 2 | 2 | 4 | 1 |
| Guinea | 1 | 1 | 2 | 1 | 2 | 2 | 4 | 1 |
| Guinea-Bissau | 1 | 1 | 2 | 1 | 2 | 2 | 4 | 1 |
| Guyana | 1 | 1 | 2 | 1 | 2 | 2 | 4 | 1 |
| Haiti | 1 | 1 | 1 | 1 | 2 | 2 | 4 | 1 |
| Honduras | 1 | 1 | 1 | 1 | 2 | 2 | 4 | 1 |
| Hungary | 1 | 1 | 1 | 1 | 1 | 1 | 1 | 1 |
| Iceland | 1 | 1 | 1 | 1 | 2 | 2 | 4 | 1 |
| India | 1 | 1 | 1 | 1 | 1 | 1 | 4 | 1 |
| Indonesia | 1 | 1 | 1 | 1 | 1 | 1 | 4 | 1 |
| Iran | 1 | 1 | 1 | 1 | 2 | 2 | 4 | 1 |
| Iraq | 1 | 1 | 1 | 1 | 2 | 2 | 4 | 1 |
| Ireland | 1 | 1 | 1 | 1 | 1 | 1 | 1 | 1 |
| Israel | 1 | 1 | 1 | 1 | 2 | 2 | 4 | 1 |
| Italy | 1 | 1 | 1 | 1 | 1 | 1 | 1 | 1 |
| Jamaica | 1 | 1 | 1 | 1 | 2 | 2 | 4 | 1 |
| Japan | 1 | 1 | 1 | 1 | 1 | 1 | 1 | 1 |
| Jordan | 1 | 1 | 1 | 1 | 2 | 2 | 4 | 1 |
| Kazakhstan | 1 | 1 | 1 | 1 | 2 | 2 | 4 | 1 |
| Kenya | 1 | 1 | 1 | 1 | 2 | 2 | 4 | 1 |
| Kiribati | 1 | 1 | 2 | 1 | 3 | 3 | 4 | 1 |
| Kosovo | 1 | 3 | 1 | 3 | 2 | 2 | 4 | 3 |
| Kuwait | 1 | 1 | 1 | 1 | 2 | 2 | 4 | 1 |
| Kyrgyz Republic | 1 | 1 | 1 | 1 | 2 | 2 | 4 | 1 |
| Laos | 1 | 1 | 2 | 1 | 2 | 2 | 4 | 1 |
| Latvia | 1 | 1 | 1 | 1 | 1 | 1 | 4 | 1 |
| Lebanon | 1 | 1 | 1 | 1 | 2 | 2 | 4 | 1 |
| Lesotho | 1 | 2 | 2 | 1 | 3 | 3 | 4 | 1 |
| Liberia | 1 | 1 | 2 | 1 | 2 | 2 | 4 | 1 |
| Libya | 1 | 1 | 1 | 1 | 2 | 2 | 4 | 1 |
| Liechtenstein | 1 | 3 | 3 | 1 | 3 | 3 | 4 | 1 |
| Lithuania | 1 | 1 | 1 | 1 | 1 | 1 | 1 | 1 |
| Luxembourg | 1 | 2 | 1 | 1 | 1 | 1 | 4 | 1 |
| Madagascar | 1 | 1 | 2 | 1 | 2 | 2 | 4 | 1 |
| Malawi | 1 | 1 | 2 | 1 | 2 | 2 | 4 | 1 |
| Malaysia | 1 | 1 | 1 | 1 | 2 | 2 | 4 | 1 |
| Maldives | 1 | 1 | 2 | 1 | 2 | 2 | 4 | 1 |
| Mali | 1 | 1 | 2 | 1 | 2 | 2 | 4 | 1 |
| Malta | 1 | 1 | 1 | 1 | 1 | 1 | 4 | 1 |
| Marshall Islands | 1 | 1 | 2 | 1 | 2 | 2 | 4 | 3 |
| Mauritania | 1 | 1 | 2 | 1 | 2 | 2 | 4 | 1 |
| Mauritius | 1 | 1 | 2 | 1 | 2 | 2 | 4 | 1 |
| Mexico | 1 | 1 | 1 | 1 | 1 | 1 | 4 | 1 |
| Micronesia, Fed. Sts. | 1 | 1 | 3 | 1 | 2 | 2 | 4 | 3 |
| Monaco | 1 | 3 | 3 | 3 | 3 | 3 | 4 | 3 |
| Mongolia | 1 | 1 | 1 | 1 | 2 | 2 | 4 | 1 |
| Montenegro | 1 | 1 | 1 | 1 | 2 | 2 | 4 | 2 |
| Montserrat | 1 | 1 | 2 | 1 | 2 | 2 | 4 | 1 |
| Morocco | 1 | 1 | 1 | 1 | 2 | 2 | 4 | 1 |
| Mozambique | 1 | 1 | 1 | 1 | 2 | 2 | 4 | 1 |
| Myanmar | 1 | 1 | 1 | 1 | 2 | 2 | 4 | 1 |
| Namibia | 1 | 2 | 1 | 1 | 3 | 3 | 4 | 1 |
| Nauru | 1 | 1 | 2 | 1 | 3 | 3 | 4 | 3 |
| Nepal | 1 | 1 | 1 | 1 | 2 | 2 | 4 | 1 |
| Netherlands | 1 | 1 | 1 | 1 | 1 | 1 | 1 | 1 |
| New Caledonia | 1 | 1 | 2 | 1 | 3 | 3 | 4 | 1 |
| New Zealand | 1 | 1 | 1 | 1 | 2 | 2 | 4 | 1 |
| Nicaragua | 1 | 1 | 1 | 1 | 2 | 2 | 4 | 1 |
| Niger | 1 | 1 | 2 | 1 | 2 | 2 | 4 | 1 |
| Nigeria | 1 | 1 | 1 | 1 | 2 | 2 | 4 | 1 |
| Norway | 1 | 1 | 1 | 1 | 1 | 1 | 1 | 1 |
| Oman | 1 | 1 | 1 | 1 | 2 | 2 | 4 | 1 |
| Pakistan | 1 | 1 | 1 | 1 | 2 | 2 | 4 | 1 |
| Palau | 1 | 1 | 2 | 3 | 2 | 2 | 4 | 3 |
| Panama | 1 | 1 | 1 | 1 | 2 | 2 | 4 | 1 |
| Papua New Guinea | 1 | 1 | 2 | 1 | 3 | 3 | 4 | 1 |
| Paraguay | 1 | 1 | 1 | 1 | 2 | 2 | 4 | 1 |
| Peru | 1 | 1 | 1 | 1 | 2 | 2 | 4 | 1 |
| Philippines | 1 | 1 | 1 | 1 | 2 | 2 | 4 | 1 |
| Poland | 1 | 1 | 1 | 1 | 1 | 1 | 1 | 1 |
| Portugal | 1 | 1 | 1 | 1 | 1 | 1 | 1 | 1 |
| Puerto Rico | 1 | 3 | 2 | 1 | 2 | 2 | 4 | 1 |
| Qatar | 1 | 1 | 1 | 1 | 2 | 2 | 4 | 1 |
| South Korea | 1 | 1 | 1 | 1 | 1 | 1 | 4 | 1 |
| Moldova | 1 | 1 | 1 | 1 | 2 | 2 | 4 | 1 |
| Romania | 1 | 1 | 1 | 1 | 1 | 1 | 1 | 1 |
| Russia | 1 | 1 | 1 | 1 | 1 | 1 | 4 | 1 |
| Rwanda | 1 | 1 | 2 | 1 | 2 | 2 | 4 | 1 |
| St. Kitts and Nevis | 1 | 1 | 2 | 1 | 2 | 2 | 4 | 1 |
| St. Lucia | 1 | 1 | 2 | 1 | 2 | 2 | 4 | 1 |
| Samoa | 1 | 1 | 2 | 1 | 3 | 3 | 4 | 1 |
| San Marino | 1 | 1 | 3 | 3 | 3 | 3 | 4 | 3 |
| Sao Tome and Principe | 1 | 1 | 2 | 1 | 2 | 2 | 4 | 1 |
| Saudi Arabia | 1 | 1 | 1 | 1 | 2 | 2 | 4 | 1 |
| Senegal | 1 | 1 | 1 | 1 | 2 | 2 | 4 | 1 |
| Serbia | 1 | 1 | 1 | 1 | 2 | 2 | 4 | 2 |
| Seychelles | 1 | 1 | 2 | 1 | 2 | 2 | 4 | 3 |
| Sierra Leone | 1 | 1 | 2 | 1 | 2 | 2 | 4 | 1 |
| Singapore | 1 | 1 | 1 | 1 | 2 | 2 | 4 | 1 |
| Sint Maarten | 1 | 1 | 2 | 3 | 2 | 2 | 4 | 3 |
| Slovakia | 1 | 1 | 1 | 1 | 1 | 1 | 1 | 1 |
| Slovenia | 1 | 1 | 1 | 1 | 1 | 1 | 1 | 1 |
| Solomon Islands | 1 | 1 | 2 | 1 | 3 | 3 | 4 | 1 |
| Somalia | 1 | 1 | 2 | 1 | 2 | 2 | 4 | 1 |
| South Africa | 1 | 2 | 1 | 1 | 1 | 1 | 4 | 1 |
| South Sudan | 1 | 1 | 2 | 3 | 2 | 2 | 4 | 1 |
| Spain | 1 | 1 | 1 | 1 | 1 | 1 | 1 | 1 |
| Sri Lanka | 1 | 1 | 1 | 1 | 2 | 2 | 4 | 1 |
| St. Vincent and the Grenadines | 1 | 1 | 2 | 1 | 2 | 2 | 4 | 1 |
| Palestine | 1 | 1 | 3 | 1 | 2 | 2 | 4 | 3 |
| Sudan | 1 | 1 | 1 | 1 | 2 | 2 | 4 | 1 |
| Suriname | 1 | 1 | 2 | 1 | 2 | 2 | 4 | 1 |
| Swaziland | 1 | 2 | 2 | 1 | 3 | 3 | 4 | 1 |
| Sweden | 1 | 1 | 1 | 1 | 1 | 1 | 1 | 1 |
| Switzerland | 1 | 1 | 1 | 1 | 1 | 1 | 4 | 1 |
| Syria | 1 | 1 | 1 | 1 | 2 | 2 | 4 | 1 |
| Macedonia | 1 | 1 | 1 | 1 | 2 | 2 | 4 | 1 |
| Tajikistan | 1 | 1 | 1 | 1 | 2 | 2 | 4 | 1 |
| Thailand | 1 | 1 | 1 | 1 | 2 | 2 | 4 | 1 |
| Timor-Leste | 1 | 1 | 2 | 1 | 3 | 3 | 4 | 1 |
| Togo | 1 | 1 | 1 | 1 | 2 | 2 | 4 | 1 |
| Tonga | 1 | 1 | 2 | 1 | 3 | 3 | 4 | 1 |
| Trinidad and Tobago | 1 | 1 | 1 | 1 | 2 | 2 | 4 | 1 |
| Tunisia | 1 | 1 | 1 | 1 | 2 | 2 | 4 | 1 |
| Turkey | 1 | 1 | 1 | 1 | 1 | 1 | 4 | 1 |
| Turkmenistan | 1 | 1 | 1 | 1 | 2 | 2 | 4 | 1 |
| Turks and Caicos Islands | 1 | 1 | 2 | 3 | 2 | 2 | 4 | 1 |
| Tuvalu | 1 | 1 | 2 | 1 | 3 | 3 | 4 | 3 |
| Tanzania | 1 | 1 | 1 | 1 | 2 | 2 | 4 | 1 |
| Uganda | 1 | 1 | 2 | 1 | 2 | 2 | 4 | 1 |
| Ukraine | 1 | 1 | 1 | 1 | 2 | 2 | 4 | 1 |
| United Arab Emirates | 1 | 1 | 1 | 1 | 2 | 2 | 4 | 1 |
| United Kingdom | 1 | 1 | 1 | 1 | 1 | 1 | 1 | 1 |
| United States | 1 | 1 | 1 | 1 | 1 | 1 | 1 | 1 |
| Uruguay | 1 | 1 | 1 | 1 | 2 | 2 | 4 | 1 |
| Uzbekistan | 1 | 1 | 1 | 1 | 2 | 2 | 4 | 1 |
| Vanuatu | 1 | 1 | 2 | 1 | 3 | 3 | 4 | 1 |
| Venezuela | 1 | 1 | 1 | 1 | 2 | 2 | 4 | 1 |
| Vietnam | 1 | 1 | 1 | 1 | 2 | 2 | 4 | 1 |
| Yemen | 1 | 1 | 1 | 1 | 2 | 2 | 4 | 1 |
| Zambia | 1 | 1 | 1 | 1 | 2 | 2 | 4 | 1 |
| Zanzibar | 1 | 3 | 2 | 3 | 2 | 2 | 4 | 3 |
| Zimbabwe | 1 | 1 | 1 | 1 | 2 | 2 | 4 | 1 |
| Taiwan | 1 | 3 | 1 | 1 | 1 | 1 | 4 | 1 |

# Industry concordance: UN ISIC to EXIOBASE

Concordances.xlsx

Sheet: ISIC to Industries

# Region concordance: EXIOBASE 3rx to EXIOBASE 3

Concordances.xlsx

Sheet: Countries

# Unbalanced countries-years

# China cropland embodied in trade

Table S 2 shows China’s cropland embodied in trade from 1995 to 2015 as well as the balance of traded cropland for EXIOBASE 3rx and the aggregated database.

Table S 2: China's cropland embodied in trade. The trade balance is the exported land minus the imported land in km2

|  | **EXIOBASE 3rx** | | | | **Aggregated Database** | | | |  | |
| --- | --- | --- | --- | --- | --- | --- | --- | --- | --- | --- |
| **Year** | **Imports (km²)** | **Exports (km²)** | **Trade balance (km²)** | **Imports as share of exports** | **Imports (km²)** | **Exports (km²)** | **Trade balance (km²)** | **Imports as share of exports** | |  |
| **1995** | 59 400 | 165 000 | 105 000 | 36 % | 63 100 | 165 000 | 102 000 | 38 % | |  |
| **1996** | 63 900 | 166 000 | 102 000 | 39 % | 69 200 | 166 000 | 96 400 | 42 % | |  |
| **1997** | 74 000 | 196 000 | 122 000 | 38 % | 78 000 | 196 000 | 118 000 | 40 % | |  |
| **1998** | 81 700 | 170 000 | 88 700 | 48 % | 86 700 | 170 000 | 83 700 | 51 % | |  |
| **1999** | 85 300 | 202 000 | 117 000 | 42 % | 85 000 | 202 000 | 117 000 | 42 % | |  |
| **2000** | 160 000 | 171 000 | 10 800 | 94 % | 159 000 | 171 000 | 12 100 | 93 % | |  |
| **2001** | 303 000 | 214 000 | -89 200 | 142 % | 327 000 | 214 000 | -113 000 | 153 % | |  |
| **2002** | 262 000 | 234 000 | -27 800 | 112 % | 293 000 | 234 000 | -58 700 | 125 % | |  |
| **2003** | 434 000 | 267 000 | -167 000 | 163 % | 469 000 | 267 000 | -202 000 | 176 % | |  |
| **2004** | 543 000 | 253 000 | -290 000 | 214 % | 590 000 | 253 000 | -337 000 | 233 % | |  |
| **2005** | 601 000 | 286 000 | -315 000 | 210 % | 651 000 | 286 000 | -365 000 | 228 % | |  |
| **2006** | 650 000 | 296 000 | -353 000 | 219 % | 694 000 | 296 000 | -398 000 | 234 % | |  |
| **2007** | 623 000 | 319 000 | -304 000 | 196 % | 670 000 | 319 000 | -351 000 | 210 % | |  |
| **2008** | 761 000 | 278 000 | -482 000 | 273 % | 797 000 | 278 000 | -519 000 | 286 % | |  |
| **2009** | 778 000 | 237 000 | -541 000 | 328 % | 812 000 | 237 000 | -575 000 | 342 % | |  |
| **2010** | 987 000 | 249 000 | -738 000 | 397 % | 1 020 000 | 249 000 | -773 000 | 411 % | |  |
| **2011** | 1 010 000 | 243 000 | -770 000 | 416 % | 1 040 000 | 243 000 | -799 000 | 428 % | |  |
| **2012** | 1 140 000 | 239 000 | -899 000 | 476 % | 1 180 000 | 239 000 | -936 000 | 491 % | |  |
| **2013** | 1 250 000 | 230 000 | -1 020 000 | 544 % | 1 210 000 | 230 000 | -984 000 | 528 % | |  |
| **2014** | 1 260 000 | 221 000 | -1 040 000 | 570 % | 1 220 000 | 221 000 | -1 000 000 | 554 % | |  |
| **2015** | 1 230 000 | 231 000 | -1 000 000 | 533 % | 1 200 000 | 231 000 | -970 000 | 520 % | |  |

# Land use results time series

SI7_results.xlsx

# Aggregation error 2015

## Exports per region

Table S 3: Regions ranked according to aggregation error score of land embodied in exports (2015). No aggregation error for the non-RoW regions. The error score is relative to the total value of the specific flow of exports. The share of total aggregation error refers to the aggregation error summed across all flows (i.e. global). The difference between databases shows the value of the flow in the aggregated database compared to that in EXIOBASE 3rx

| **Region** | **Total land area of flow (km²)** | **Share of global land area (km²)** | **Aggregation error (km²)** | **error score (ε)** | **Share of total aggregation error** | **Difference between databases (100% is equal to no difference)** |
| --- | --- | --- | --- | --- | --- | --- |
| WA | 1 580 000 | 7.2 % | 1 630 000 | 1.03 | 47.9 % | 121.8 % |
| WM | 175 000 | 0.8 % | 127 000 | 0.73 | 3.7 % | 95.2 % |
| WL | 1 500 000 | 6.9 % | 568 000 | 0.38 | 16.7 % | 84.9 % |
| WF | 2 870 000 | 13.1 % | 925 000 | 0.32 | 27.1 % | 99.4 % |
| WE | 477 000 | 2.2 % | 154 000 | 0.32 | 4.5 % | 85.8 % |

## Imports per region and product

Table S 4: Land embodied in imports and associated aggregation errors by top contributing products and regions sorted by aggregation error in km ^2^(2015). The error score is relative to the total value of the specific flow of imports. The share of total aggregation error refers to the aggregation error summed across all flows (i.e. global). The difference between databases shows the value of the flow in the aggregated database compared to that in EXIOBASE 3rx

| **Region** | **Product** | **Total land area of flow (km²)** | **Share of global land area (km²)** | **Aggregation error (km²)** | **error score (ε)** | **Share of total aggregation error** | **Difference between databases (100% is equal to no difference)** |
| --- | --- | --- | --- | --- | --- | --- | --- |
| TW | Products of forestry, logging and related services (02) | 845 000 | 3.9 % | 218 000 | 0.26 | 6.4 % | 74 % |
| CN | Products of forestry, logging and related services (02) | 3 940 000 | 18.0 % | 122 000 | 0.03 | 3.6 % | 98 % |
| CN | Oil seeds | 870 000 | 4.0 % | 98 800 | 0.11 | 2.9 % | 92 % |
| IN | Products of forestry, logging and related services (02) | 285 000 | 1.3 % | 85 100 | 0.30 | 2.5 % | 118 % |
| CN | Copper ores and concentrates | 71 800 | 0.3 % | 63 800 | 0.89 | 1.9 % | 11 % |
| CN | Hotel and restaurant services (55) | 5 390 | 0.0 % | 48 000 | 8.91 | 1.4 % | 991 % |
| WM | Wheat | 297 000 | 1.4 % | 41 600 | 0.14 | 1.2 % | 86 % |
| RU | Hotel and restaurant services (55) | 63 200 | 0.3 % | 40 900 | 0.65 | 1.2 % | 47 % |
| WM | Cereal grains nec | 201 000 | 0.9 % | 39 300 | 0.20 | 1.2 % | 81 % |
| JP | Meat animals nec | 5 940 | 0.0 % | 36 800 | 6.19 | 1.1 % | 714 % |
| CN | Meat animals nec | 56 800 | 0.3 % | 35 100 | 0.62 | 1.0 % | 38 % |
| US | Food products nec | 107 000 | 0.5 % | 34 300 | 0.32 | 1.0 % | 132 % |
| CN | Cattle | 300 000 | 1.4 % | 28 000 | 0.09 | 0.8 % | 107 % |
| CN | Chemicals nec | 57 800 | 0.3 % | 25 900 | 0.45 | 0.8 % | 67 % |
| CN | Wood and products of wood and cork (except furniture); articles of straw and plaiting materials (20) | 180 000 | 0.8 % | 24 400 | 0.14 | 0.7 % | 87 % |
| WA | Products of forestry, logging and related services (02) | 124 000 | 0.6 % | 23 400 | 0.19 | 0.7 % | 119 % |
| WM | Oil seeds | 80 000 | 0.4 % | 23 000 | 0.29 | 0.7 % | 102 % |
| FR | Meat animals nec | 1 310 | 0.0 % | 22 800 | 17.43 | 0.7 % | 1840 % |
| BR | Products of forestry, logging and related services (02) | 32 300 | 0.1 % | 21 900 | 0.68 | 0.6 % | 33 % |
| TW | Copper ores and concentrates | 23 000 | 0.1 % | 21 700 | 0.94 | 0.6 % | 6 % |

## Imports per region, product and partner

Table S 5: Top 20 flows ranked according to aggregation error (absolute value) of land embodied in imports for products and regions (2015). The error score is relative to the total value of the specific flow of imports. The share of total aggregation error refers to the aggregation error summed across all flows (i.e. global). The difference between databases shows the value of the flow in the aggregated database compared to that in EXIOBASE 3rx

| **Region** | **Product** | **Partner** | **Total land area of flow (km²)** | **Share of global land area (km²)** | **Aggregation error (km²)** | **error score (ε)** | **Share of total aggregation error** | **Difference between databases (100% is equal to no difference)** |
| --- | --- | --- | --- | --- | --- | --- | --- | --- |
| TW | Products of forestry, logging and related services (02) | WA | 155 000 | 0.7 % | 116 000 | 0.75 | 3.4 % | 25 % |
| TW | Products of forestry, logging and related services (02) | WF | 288 000 | 1.3 % | 89 100 | 0.31 | 2.6 % | 69 % |
| CN | Oil seeds | WL | 163 000 | 0.7 % | 69 900 | 0.43 | 2.1 % | 57 % |
| CN | Products of forestry, logging and related services (02) | WL | 186 000 | 0.9 % | 66 100 | 0.36 | 1.9 % | 64 % |
| CN | Copper ores and concentrates | WA | 56 500 | 0.3 % | 50 700 | 0.90 | 1.5 % | 10 % |
| IN | Products of forestry, logging and related services (02) | WF | 32 200 | 0.1 % | 49 400 | 1.53 | 1.4 % | 253 % |
| CN | Hotel and restaurant services (55) | WA | 3 060 | 0.0 % | 48 000 | 15.7 | 1.4 % | 1670 % |
| RU | Hotel and restaurant services (55) | WA | 55 500 | 0.3 % | 37 200 | 0.67 | 1.1 % | 33 % |
| JP | Meat animals nec | WA | 1 080 | 0.0 % | 36 600 | 33.8 | 1.1 % | 3480 % |
| CN | Products of forestry, logging and related services (02) | WA | 119 000 | 0.5 % | 34 600 | 0.29 | 1.0 % | 71 % |
| WM | Wheat | WA | 37 700 | 0.2 % | 32 500 | 0.86 | 1.0 % | 14 % |
| CN | Meat animals nec | WA | 42 000 | 0.2 % | 31 300 | 0.75 | 0.9 % | 26 % |
| WM | Cereal grains nec | WA | 30 300 | 0.1 % | 25 900 | 0.86 | 0.8 % | 14 % |
| US | Food products nec | WL | 14 800 | 0.1 % | 23 900 | 1.61 | 0.7 % | 261 % |
| CN | Cattle | WA | 17 400 | 0.1 % | 23 800 | 1.36 | 0.7 % | 236 % |
| BR | Products of forestry, logging and related services (02) | WL | 31 600 | 0.1 % | 21 900 | 0.69 | 0.6 % | 31 % |
| CN | Chemicals nec | WM | 37 200 | 0.2 % | 21 400 | 0.58 | 0.6 % | 42 % |
| FR | Meat animals nec | WA | 99 | 0.0 % | 21 400 | 217 | 0.6 % | 21800 % |
| CN | Products of forestry, logging and related services (02) | WF | 1 050 000 | 4.8 % | 20 600 | 0.02 | 0.6 % | 102 % |
| TW | Copper ores and concentrates | WA | 20 800 | 0.1 % | 19 900 | 0.95 | 0.6 % | 5 % |

# Aggregation effect all years

The tables underneath show the effect of regional aggregation on land embodied in trade across all years.

## Exports per region

Table S 6: Regions ranked according to aggregation error score of land embodied in exports. No aggregation error for the non-RoW regions. The error score is relative to the total value of the specific flow of exports. The share of total aggregation error refers to the aggregation error summed across all flows (i.e. global). The difference between databases shows the value of the flow in the aggregated database compared to that in EXIOBASE 3rx

| **Region** | **Total land area of flow (km²)** | **Share of global land area (km²)** | **Aggregation error (km²)** | **error score (ε)** | **Share of total aggregation error** | **Difference between databases (100% is equal to no difference)** |
| --- | --- | --- | --- | --- | --- | --- |
| WA | 37 600 000 | 10.8 % | 40 700 000 | 1.08 | 45.6 % | 129 % |
| WM | 3 360 000 | 1.0 % | 2 490 000 | 0.74 | 2.8 % | 113 % |
| WF | 54 400 000 | 15.6 % | 33 400 000 | 0.61 | 37.4 % | 95 % |
| WL | 26 100 000 | 7.5 % | 10 900 000 | 0.42 | 12.1 % | 100 % |
| WE | 8 540 000 | 2.4 % | 1 830 000 | 0.21 | 2.0 % | 90 % |

## Imports per region

Table S 7: Regions ranked according to aggregation error score of land embodied in imports. The error score is relative to the total value of the specific flow of imports. The share of total aggregation error refers to the aggregation error summed across all flows (i.e. global). The difference between databases shows the value of the flow in the aggregated database compared to that in EXIOBASE 3rx

| **Region** | **Total land area of flow (km²)** | **Share of global land area (km²)** | **Aggregation error (km²)** | **error score (ε)** | **Share of total aggregation error** | **Difference between databases (100% is equal to no difference)** |
| --- | --- | --- | --- | --- | --- | --- |
| AU | 1 710 000 | 0.5 % | 1 480 000 | 0.87 | 1.7 % | 179 % |
| MT | 69 300 | 0.0 % | 58 500 | 0.84 | 0.1 % | 172 % |
| PT | 6 400 000 | 1.8 % | 4 030 000 | 0.63 | 4.5 % | 45 % |
| RU | 10 200 000 | 2.9 % | 5 410 000 | 0.53 | 6.1 % | 54 % |
| IN | 7 780 000 | 2.2 % | 3 900 000 | 0.50 | 4.4 % | 129 % |
| ZA | 2 440 000 | 0.7 % | 1 180 000 | 0.48 | 1.3 % | 80 % |
| SI | 454 000 | 0.1 % | 215 000 | 0.47 | 0.2 % | 68 % |
| FR | 11 800 000 | 3.4 % | 5 170 000 | 0.44 | 5.8 % | 102 % |
| BR | 4 160 000 | 1.2 % | 1 750 000 | 0.42 | 2.0 % | 81 % |
| CH | 1 110 000 | 0.3 % | 461 000 | 0.41 | 0.5 % | 114 % |
| LU | 424 000 | 0.1 % | 167 000 | 0.39 | 0.2 % | 105 % |
| ES | 5 760 000 | 1.6 % | 2 200 000 | 0.38 | 2.5 % | 115 % |
| IT | 10 400 000 | 3.0 % | 3 880 000 | 0.37 | 4.3 % | 103 % |
| IE | 714 000 | 0.2 % | 251 000 | 0.35 | 0.3 % | 126 % |
| GB | 9 090 000 | 2.6 % | 3 170 000 | 0.35 | 3.5 % | 121 % |
| TW | 10 300 000 | 3.0 % | 3 390 000 | 0.33 | 3.8 % | 100 % |
| BE | 4 120 000 | 1.2 % | 1 330 000 | 0.32 | 1.5 % | 98 % |
| TR | 5 350 000 | 1.5 % | 1 610 000 | 0.30 | 1.8 % | 90 % |
| DE | 11 800 000 | 3.4 % | 3 540 000 | 0.30 | 4.0 % | 109 % |
| DK | 1 400 000 | 0.4 % | 414 000 | 0.30 | 0.5 % | 111 % |
| RO | 563 000 | 0.2 % | 165 000 | 0.29 | 0.2 % | 92 % |
| ID | 6 360 000 | 1.8 % | 1 740 000 | 0.27 | 1.9 % | 122 % |
| NL | 6 330 000 | 1.8 % | 1 680 000 | 0.27 | 1.9 % | 116 % |
| HR | 254 000 | 0.1 % | 67 200 | 0.26 | 0.1 % | 107 % |
| LT | 416 000 | 0.1 % | 108 000 | 0.26 | 0.1 % | 83 % |
| GR | 1 470 000 | 0.4 % | 380 000 | 0.26 | 0.4 % | 100 % |
| WE | 2 780 000 | 0.8 % | 705 000 | 0.25 | 0.8 % | 84 % |
| CZ | 735 000 | 0.2 % | 180 000 | 0.25 | 0.2 % | 96 % |
| US | 36 200 000 | 10.4 % | 8 850 000 | 0.24 | 9.9 % | 118 % |
| CY | 140 000 | 0.0 % | 33 700 | 0.24 | 0.0 % | 103 % |
| NO | 1 650 000 | 0.5 % | 391 000 | 0.24 | 0.4 % | 101 % |
| JP | 25 900 000 | 7.4 % | 5 810 000 | 0.22 | 6.5 % | 118 % |
| BG | 241 000 | 0.1 % | 49 800 | 0.21 | 0.1 % | 103 % |
| CN | 61 000 000 | 17.4 % | 12 500 000 | 0.20 | 14.0 % | 92 % |
| HU | 650 000 | 0.2 % | 128 000 | 0.20 | 0.1 % | 97 % |
| WM | 24 300 000 | 6.9 % | 4 700 000 | 0.19 | 5.3 % | 99 % |
| CA | 4 980 000 | 1.4 % | 953 000 | 0.19 | 1.1 % | 114 % |
| AT | 1 560 000 | 0.4 % | 297 000 | 0.19 | 0.3 % | 106 % |
| PL | 1 620 000 | 0.5 % | 302 000 | 0.19 | 0.3 % | 97 % |
| SE | 2 930 000 | 0.8 % | 540 000 | 0.18 | 0.6 % | 113 % |
| SK | 353 000 | 0.1 % | 58 200 | 0.16 | 0.1 % | 105 % |
| LV | 460 000 | 0.1 % | 70 700 | 0.15 | 0.1 % | 87 % |
| KR | 13 000 000 | 3.7 % | 1 870 000 | 0.14 | 2.1 % | 102 % |
| WF | 7 640 000 | 2.2 % | 935 000 | 0.12 | 1.0 % | 104 % |
| MX | 5 890 000 | 1.7 % | 613 000 | 0.10 | 0.7 % | 103 % |
| WL | 6 470 000 | 1.8 % | 652 000 | 0.10 | 0.7 % | 104 % |
| EE | 478 000 | 0.1 % | 46 800 | 0.10 | 0.1 % | 96 % |
| WA | 23 500 000 | 6.7 % | 1 650 000 | 0.07 | 1.8 % | 99 % |
| FI | 6 330 000 | 1.8 % | 242 000 | 0.04 | 0.3 % | 100 % |

## Imports/exports per product

Table S 8: Top 20 products ranked according to aggregation error (absolute value) of land embodied in trade. The error score is relative to the total value of the specific flow of imports. The share of total aggregation error refers to the aggregation error summed across all flows (i.e. global). The difference between databases shows the value of the flow in the aggregated database compared to that in EXIOBASE 3rx

| **Product** | **Total land area of flow (km²)** | **Share of global land area (km²)** | **Aggregation error (km²)** | **error score (ε)** | **Share of total aggregation error** | **Difference between databases (100% is equal to no difference)** |
| --- | --- | --- | --- | --- | --- | --- |
| Products of forestry, logging and related services (02) | 96 300 000 | 27.5 % | 23 100 000 | 0.24 | 25.9 % | 88 % |
| Meat animals nec | 9 720 000 | 2.8 % | 5 860 000 | 0.60 | 6.6 % | 117 % |
| Hotel and restaurant services (55) | 7 560 000 | 2.2 % | 5 430 000 | 0.72 | 6.1 % | 137 % |
| Products of meat cattle | 29 100 000 | 8.3 % | 4 760 000 | 0.16 | 5.3 % | 102 % |
| Food products nec | 13 000 000 | 3.7 % | 2 760 000 | 0.21 | 3.1 % | 107 % |
| Wood and products of wood and cork (except furniture); articles of straw and plaiting materials (20) | 22 200 000 | 6.4 % | 2 740 000 | 0.12 | 3.1 % | 100 % |
| Crude petroleum and services related to crude oil extraction, excluding surveying | 2 660 000 | 0.8 % | 2 160 000 | 0.81 | 2.4 % | 128 % |
| Oil seeds | 22 900 000 | 6.6 % | 2 100 000 | 0.09 | 2.3 % | 100 % |
| Wheat | 16 800 000 | 4.8 % | 1 970 000 | 0.12 | 2.2 % | 90 % |
| Vegetables, fruit, nuts | 9 270 000 | 2.7 % | 1 940 000 | 0.21 | 2.2 % | 110 % |
| Chemicals nec | 5 750 000 | 1.6 % | 1 880 000 | 0.33 | 2.1 % | 108 % |
| Copper ores and concentrates | 1 840 000 | 0.5 % | 1 720 000 | 0.93 | 1.9 % | 14 % |
| Construction work (45) | 1 950 000 | 0.6 % | 1 390 000 | 0.71 | 1.6 % | 136 % |
| Other business services (74) | 1 370 000 | 0.4 % | 1 320 000 | 0.97 | 1.5 % | 165 % |
| Real estate services (70) | 1 220 000 | 0.3 % | 1 270 000 | 1.04 | 1.4 % | 182 % |
| Cereal grains nec | 10 700 000 | 3.1 % | 1 250 000 | 0.12 | 1.4 % | 94 % |
| Cattle | 15 000 000 | 4.3 % | 965 000 | 0.06 | 1.1 % | 101 % |
| Wholesale trade and commission trade services, except of motor vehicles and motorcycles (51) | 797 000 | 0.2 % | 944 000 | 1.18 | 1.1 % | 172 % |
| Fish products | 2 500 000 | 0.7 % | 899 000 | 0.36 | 1.0 % | 124 % |
| Crops nec | 7 230 000 | 2.1 % | 889 000 | 0.12 | 1.0 % | 104 % |

## Exports per region and product

Table S 9: Top 20 flows ranked according to aggregation error (absolute value) of land embodied in exports for products and regions. The error score is relative to the total value of the specific flow of exports. The share of total aggregation error refers to the aggregation error summed across all flows (i.e. global). The difference between databases shows the value of the flow in the aggregated database compared to that in EXIOBASE 3rx

| **Region** | **Product** | **Total land area of flow (km²)** | **Share of global land area (km²)** | **Aggregation error (km²)** | **error score (ε)** | **Share of total aggregation error** | **Difference between databases (100% is equal to no difference)** |
| --- | --- | --- | --- | --- | --- | --- | --- |
| WF | Products of forestry, logging and related services (02) | 24 800 000 | 7.1 % | 16 300 000 | 0.66 | 18.2 % | 165 % |
| WA | Hotel and restaurant services (55) | 2 310 000 | 0.7 % | 4 320 000 | 1.87 | 4.8 % | 57 % |
| WA | Meat animals nec | 2 270 000 | 0.6 % | 3 960 000 | 1.74 | 4.4 % | 63 % |
| WA | Products of forestry, logging and related services (02) | 8 530 000 | 2.4 % | 3 510 000 | 0.41 | 3.9 % | 106 % |
| WL | Products of forestry, logging and related services (02) | 6 870 000 | 2.0 % | 3 080 000 | 0.45 | 3.4 % | 121 % |
| WA | Products of meat cattle | 1 440 000 | 0.4 % | 2 890 000 | 2.01 | 3.2 % | 53 % |
| WA | Copper ores and concentrates | 1 690 000 | 0.5 % | 1 610 000 | 0.95 | 1.8 % | 900 % |
| WF | Crude petroleum and services related to crude oil extraction, excluding surveying | 1 620 000 | 0.5 % | 1 490 000 | 0.92 | 1.7 % | 60 % |
| WA | Wheat | 2 760 000 | 0.8 % | 1 400 000 | 0.51 | 1.6 % | 196 % |
| WF | Meat animals nec | 3 410 000 | 1.0 % | 1 400 000 | 0.41 | 1.6 % | 93 % |
| WF | Wood and products of wood and cork (except furniture); articles of straw and plaiting materials (20) | 1 380 000 | 0.4 % | 1 240 000 | 0.90 | 1.4 % | 78 % |
| WA | Food products nec | 1 310 000 | 0.4 % | 1 190 000 | 0.91 | 1.3 % | 83 % |
| WL | Products of meat cattle | 3 250 000 | 0.9 % | 1 150 000 | 0.35 | 1.3 % | 106 % |
| WL | Wood and products of wood and cork (except furniture); articles of straw and plaiting materials (20) | 3 090 000 | 0.9 % | 1 050 000 | 0.34 | 1.2 % | 114 % |
| WA | Other business services (74) | 368 000 | 0.1 % | 936 000 | 2.54 | 1.0 % | 37 % |
| WA | Real estate services (70) | 248 000 | 0.1 % | 895 000 | 3.61 | 1.0 % | 24 % |
| WF | Oil seeds | 2 150 000 | 0.6 % | 879 000 | 0.41 | 1.0 % | 82 % |
| WF | Vegetables, fruit, nuts | 2 700 000 | 0.8 % | 861 000 | 0.32 | 1.0 % | 83 % |
| WL | Oil seeds | 3 050 000 | 0.9 % | 740 000 | 0.24 | 0.8 % | 113 % |
| WA | Construction work (45) | 368 000 | 0.1 % | 730 000 | 1.98 | 0.8 % | 46 % |

## Imports per region and product

Table S 10: Top 20 flows ranked according to aggregation error (absolute value) of land embodied in imports for products and regions. The error score is relative to the total value of the specific flow of exports. The share of total aggregation error refers to the aggregation error summed across all flows (i.e. global). The difference between databases shows the value of the flow in the aggregated database compared to that in EXIOBASE 3rx

| **Region** | **Product** | **Total land area of flow (km²)** | **Share of global land area (km²)** | **Aggregation error (km²)** | **error score (ε)** | **Share of total aggregation error** | **Difference between databases (100% is equal to no difference)** |
| --- | --- | --- | --- | --- | --- | --- | --- |
| CN | Products of forestry, logging and related services (02) | 37 600 000 | 10.7 % | 5 330 000 | 0.14 | 6.0 % | 115 % |
| PT | Products of forestry, logging and related services (02) | 4 630 000 | 1.3 % | 3 490 000 | 0.75 | 3.9 % | 394 % |
| IN | Products of forestry, logging and related services (02) | 3 900 000 | 1.1 % | 2 620 000 | 0.67 | 2.9 % | 64 % |
| FR | Products of forestry, logging and related services (02) | 3 610 000 | 1.0 % | 1 760 000 | 0.49 | 2.0 % | 183 % |
| WM | Meat animals nec | 5 230 000 | 1.5 % | 1 430 000 | 0.27 | 1.6 % | 106 % |
| RU | Hotel and restaurant services (55) | 1 720 000 | 0.5 % | 1 330 000 | 0.77 | 1.5 % | 328 % |
| IT | Products of forestry, logging and related services (02) | 2 880 000 | 0.8 % | 1 300 000 | 0.45 | 1.5 % | 167 % |
| CN | Copper ores and concentrates | 1 360 000 | 0.4 % | 1 280 000 | 0.94 | 1.4 % | 1669 % |
| JP | Products of forestry, logging and related services (02) | 8 000 000 | 2.3 % | 971 000 | 0.12 | 1.1 % | 96 % |
| US | Products of forestry, logging and related services (02) | 2 540 000 | 0.7 % | 946 000 | 0.37 | 1.1 % | 91 % |
| RU | Products of meat cattle | 2 040 000 | 0.6 % | 944 000 | 0.46 | 1.1 % | 177 % |
| CN | Hotel and restaurant services (55) | 262 000 | 0.1 % | 887 000 | 3.39 | 1.0 % | 23 % |
| US | Meat animals nec | 514 000 | 0.1 % | 848 000 | 1.65 | 1.0 % | 41 % |
| CN | Meat animals nec | 990 000 | 0.3 % | 752 000 | 0.76 | 0.8 % | 181 % |
| TW | Products of meat cattle | 651 000 | 0.2 % | 715 000 | 1.10 | 0.8 % | 48 % |
| US | Products of meat cattle | 5 820 000 | 1.7 % | 683 000 | 0.12 | 0.8 % | 90 % |
| TR | Products of forestry, logging and related services (02) | 1 770 000 | 0.5 % | 647 000 | 0.37 | 0.7 % | 145 % |
| BR | Products of forestry, logging and related services (02) | 808 000 | 0.2 % | 637 000 | 0.79 | 0.7 % | 421 % |
| DE | Products of forestry, logging and related services (02) | 2 850 000 | 0.8 % | 612 000 | 0.22 | 0.7 % | 118 % |
| JP | Hotel and restaurant services (55) | 413 000 | 0.1 % | 610 000 | 1.48 | 0.7 % | 40 % |

## Imports/exports per region, product and partner

Table S 11: Top 20 flows ranked according to aggregation effect error (absolute value) of land embodied in imports for products, regions and origins. The error score is relative to the total value of the specific flow of exports. The share of total aggregation error refers to the aggregation error summed across all flows (i.e. global). The difference between databases shows the value of the flow in the aggregated database compared to that in EXIOBASE 3rx

| **Region** | **Product** | **Partner** | **Total land area of flow (km²)** | **Share of global land area (km²)** | **Aggregation error (km²)** | **error score (ε)** | **Share of total aggregation error** | **Difference between databases (100% is equal to no difference)** |
| --- | --- | --- | --- | --- | --- | --- | --- | --- |
| WF | Products of forestry, logging and related services (02) | CN | 7 920 000 | 2.3 % | 3 800 000 | 0.48 | 4.3 % | 53 % |
| WF | Products of forestry, logging and related services (02) | PT | 4 330 000 | 1.2 % | 3 470 000 | 0.80 | 3.9 % | 20 % |
| WF | Products of forestry, logging and related services (02) | IN | 947 000 | 0.3 % | 1 850 000 | 1.96 | 2.1 % | 294 % |
| WF | Products of forestry, logging and related services (02) | FR | 3 110 000 | 0.9 % | 1 640 000 | 0.53 | 1.8 % | 51 % |
| WA | Hotel and restaurant services (55) | RU | 1 500 000 | 0.4 % | 1 250 000 | 0.83 | 1.4 % | 17 % |
| WA | Copper ores and concentrates | CN | 1 280 000 | 0.4 % | 1 220 000 | 0.95 | 1.4 % | 5 % |
| WF | Products of forestry, logging and related services (02) | IT | 1 870 000 | 0.5 % | 1 140 000 | 0.61 | 1.3 % | 43 % |
| WA | Hotel and restaurant services (55) | CN | 22 600 | 0.0 % | 887 000 | 39.25 | 1.0 % | 4025 % |
| WL | Products of forestry, logging and related services (02) | CN | 1 560 000 | 0.4 % | 812 000 | 0.52 | 0.9 % | 51 % |
| WA | Products of meat cattle | RU | 830 000 | 0.2 % | 806 000 | 0.97 | 0.9 % | 3 % |
| WA | Products of forestry, logging and related services (02) | JP | 1 470 000 | 0.4 % | 749 000 | 0.51 | 0.8 % | 121 % |
| WF | Meat animals nec | WM | 2 470 000 | 0.7 % | 740 000 | 0.30 | 0.8 % | 111 % |
| WA | Meat animals nec | CN | 863 000 | 0.2 % | 716 000 | 0.83 | 0.8 % | 52 % |
| WA | Products of forestry, logging and related services (02) | CN | 2 470 000 | 0.7 % | 710 000 | 0.29 | 0.8 % | 82 % |
| WA | Products of meat cattle | TW | 74 700 | 0.0 % | 687 000 | 9.20 | 0.8 % | 1020 % |
| WA | Meat animals nec | WM | 746 000 | 0.2 % | 674 000 | 0.90 | 0.8 % | 29 % |
| WL | Products of forestry, logging and related services (02) | BR | 785 000 | 0.2 % | 626 000 | 0.80 | 0.7 % | 20 % |
| WA | Meat animals nec | US | 54 600 | 0.0 % | 625 000 | 11.46 | 0.7 % | 1246 % |
| WA | Hotel and restaurant services (55) | JP | 128 000 | 0.0 % | 605 000 | 4.74 | 0.7 % | 574 % |
| WA | Products of meat cattle | ID | 14 500 | 0.0 % | 603 000 | 41.62 | 0.7 % | 4262 % |

# Land footprints per capita for land use types

See SI7_results.xlsx for the underlying values behind the maps below:


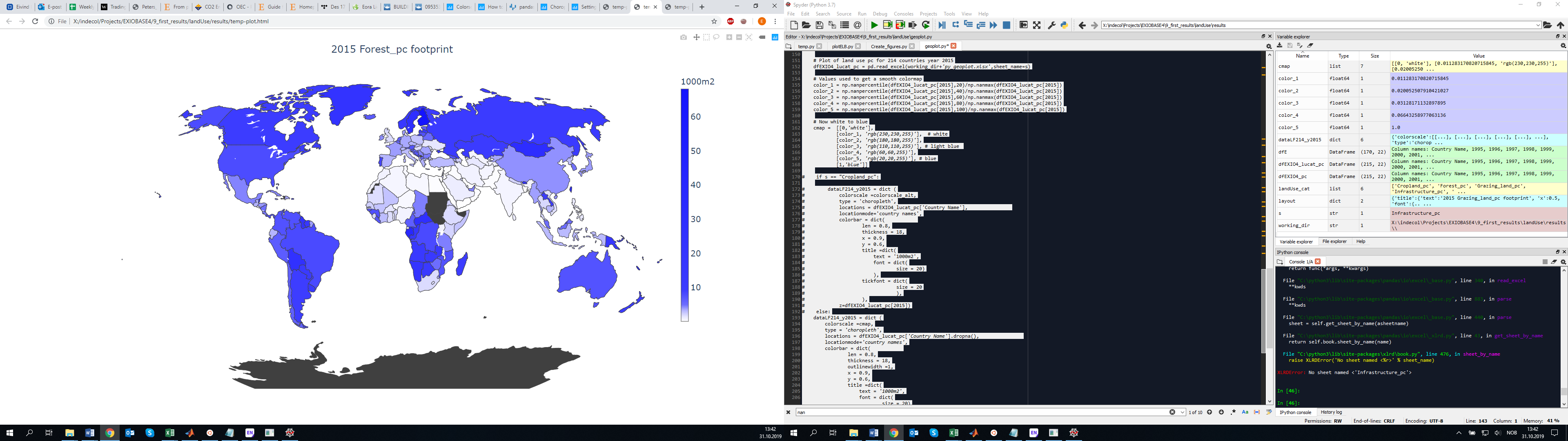


Figure S 3: Map of forestland footprints per capita for year 2015 for 214 countries. Unbalanced countries in black (Comoros, Haiti, Liechtenstein, South Sudan and Sudan)


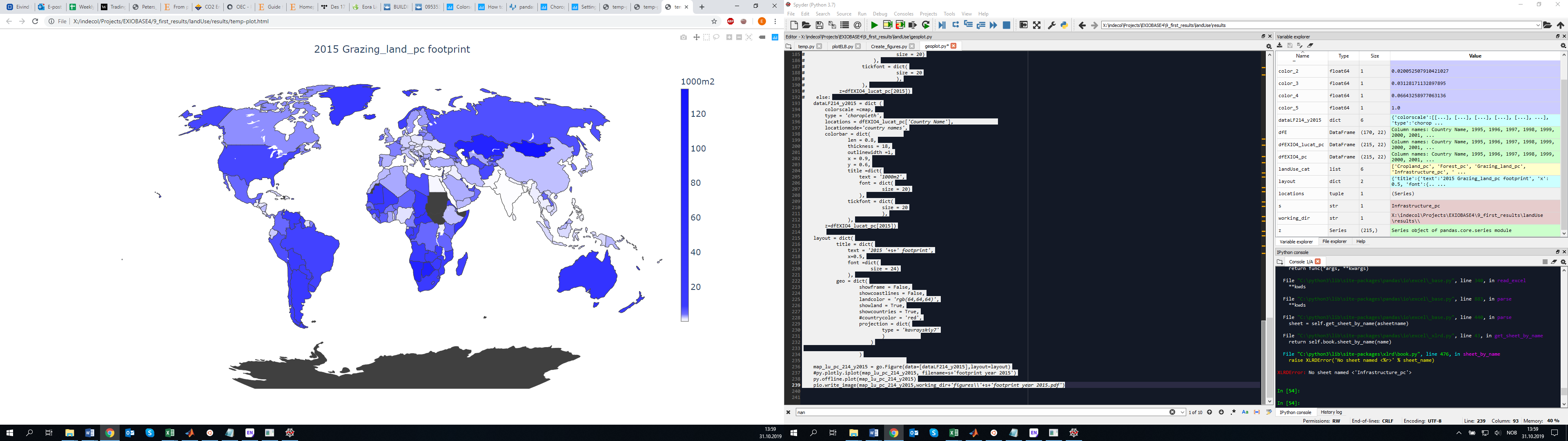


Figure S 4: Map of grazing land footprints per capita for year 2015 for 214 countries. Unbalanced countries in black (Comoros, Haiti, Liechtenstein, South Sudan and Sudan)


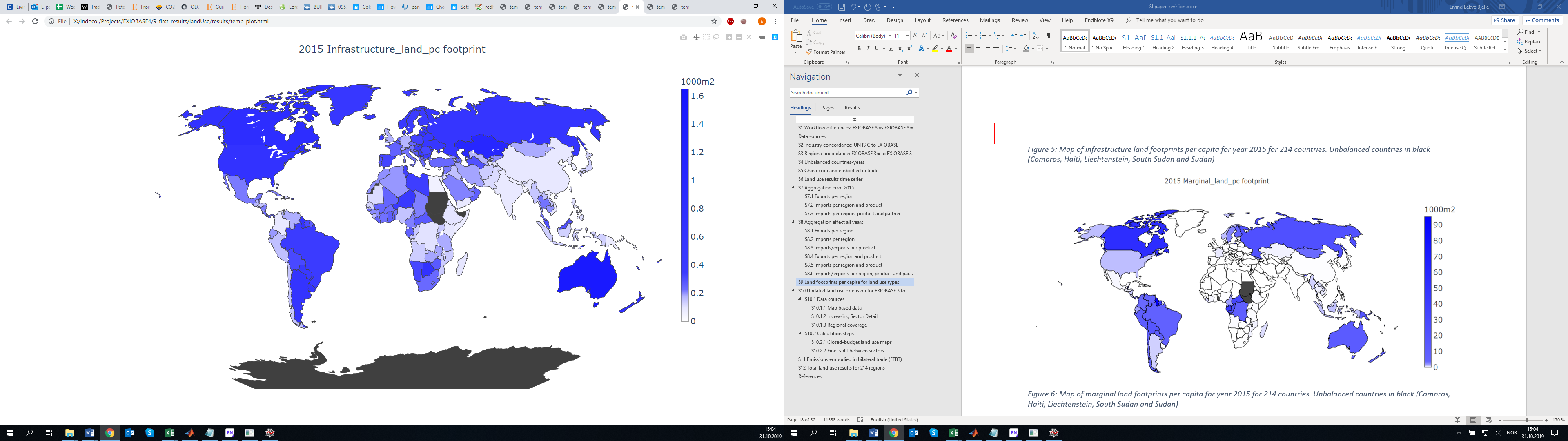


Figure S 5: Map of infrastructure land footprints per capita for year 2015 for 214 countries. Unbalanced countries in black (Comoros, Haiti, Liechtenstein, South Sudan and Sudan)


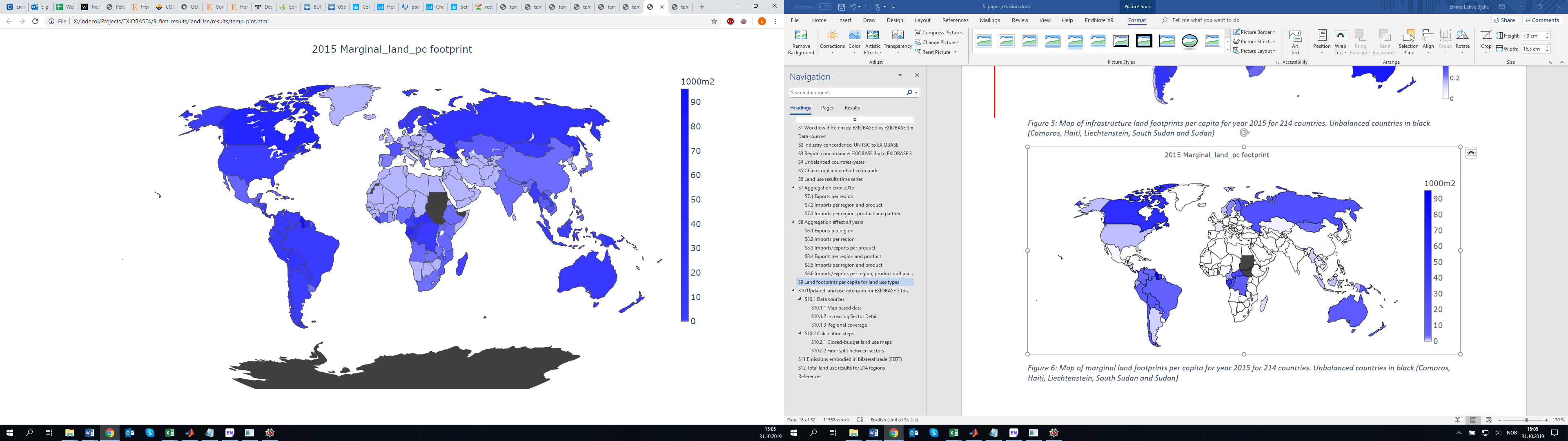


Figure S 6: Map of marginal land footprints per capita for year 2015 for 214 countries. Unbalanced countries in black (Comoros, Haiti, Liechtenstein, South Sudan and Sudan)


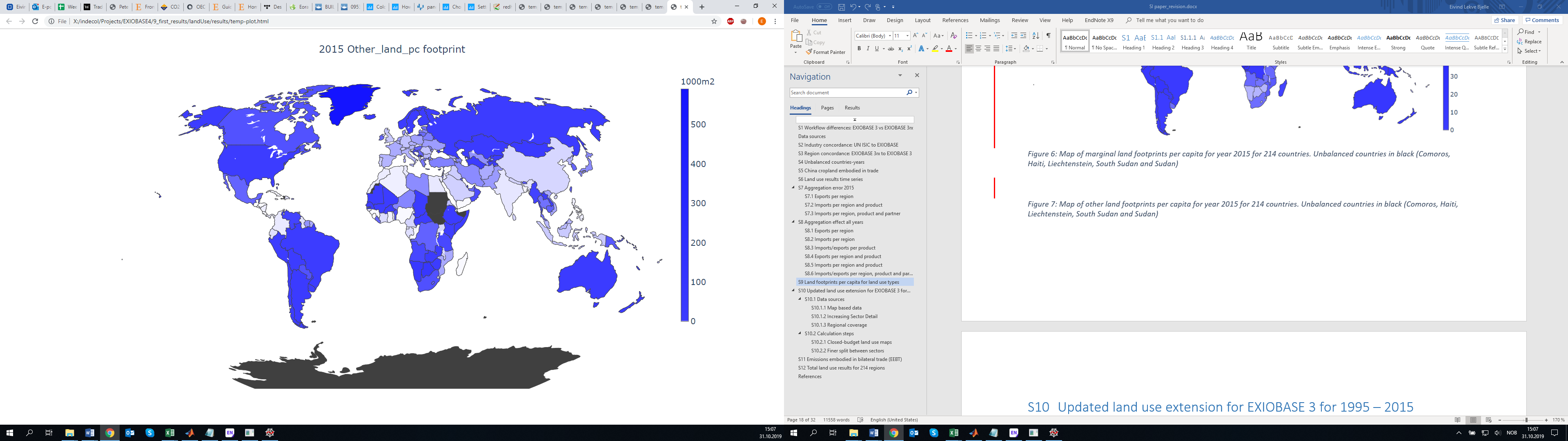


Figure S 7: Map of other land footprints per capita for year 2015 for 214 countries. Unbalanced countries in black (Comoros, Haiti, Liechtenstein, South Sudan and Sudan)

# Updated land use extension for EXIOBASE 3 for 1995–2015

Thomas Kastner, Nina Eisenmenger, Sarah Matej, Christoph Plutzar, Michaela C. Theurl, Karl-Heinz Erb

Land-use data are available at two aggregation levels and their use is free for scientific purposes. The aggregate data represents the sum of land categories allocated to the corresponding EXIOBASE sectors, and are part of the EXIOBASE 3rx download. Disaggregated data (e.g. country level or level of individual land-use categories), or more detailed data, are available upon request from the Institute of Social Ecology Vienna.

Contact: [thomas.kastner@senckenberg.de](mailto:thomas.kastner@senckenberg.de); [karlheinz.erb@boku.ac.at](mailto:karlheinz.erb@boku.ac.at)

To obtain land use data at the sectoral resolution of EXIOBASE we followed a two-step procedure:

- In a first step, we created spatially-explicit maps for major land cover types based on publicly available state-of-the-art datasets. The data were harmonized following a closed-budget mapping approach (Erb et al., 2007), i.e. the sum of all layers will add up to 100% or the available land area for each specific grid cell.
- In a second step, we utilized information from census statistics (FAOSTAT) to further disaggregate the data to closely match the EXIOBASE sector classification (in table format).

Here we list the underlying data sources before describing the calculation steps in detail.

## Data sources

### Map based data

Map based data used for the calculation draws on the following sources: data from the most resent version of the HYDE project (Klein Goldewijk et al., 2017); a recently published land-cover product from the European Space Agency, available for the years 1992 – 2015 (<http://maps.elie.ucl.ac.be/CCI/viewer/>). Data on the extent of areas without signs of human land use were taken from a recent study on intact forest landscapes (Potapov et al., 2017) and from the human footprint mapping project (Venter et al., 2016). Table 1 shows the layers created, summarizes the respective data sources for each layer and show the land use allocated to each of the layers. A land mask indicating for each cell the total amount of land area was taken from Klein Goldewijk et al. (2017). We complied the dataset in 5-arcminute resolution and converted maps with a finer spatial resolution to this resolution.

### Increasing Sector Detail

For the detailed split of the land use data to match the EXIOBASE sector resolution the following data sources were used: FAOSTAT data (<http://www.fao.org/faostat/en/#data>, last accessed October 26, 2018); countries classified as advanced economies according to the international monetary fund (as described in the land SI in Stadler et al. (2018); UNstat data for wood fuel and charcoal production and consumption (<http://data.un.org/>, last accessed October 22, 2018).

Table S 12: Spatially-explicit data layers constructed for the land-use dataset with corresponding data sources and the land uses allocated to them. Sources are: HYDE: (Klein Goldewijk et al., 2017), ESA: <http://maps.elie.ucl.ac.be/CCI/viewer/>, HFP: (Venter et al., 2016), IFL: (Potapov et al., 2017)

| **Layer** | **Data sources** | **Land uses allocated** |
| --- | --- | --- |
| Infrastructure Land | Built-up land layer and cropland layer from *HYDE* | Infrastructure |
| Cropland | Cropland layer from *HYDE* | Crop production |
| Non-productive land | *ESA* layers 200, 201, 202, 220 | - |
| Wilderness core | *HFP* and *IFL* data | - |
| Wilderness periphery | *HFP* and *IFL* data | - |
| Closed forests | *ESA* layers 50, 60, 61, 70, 71, 80, 81, 90, 160, 170 | Forestry incl. wood fuel |
| Open Forests | *ESA* layers 30, 40, 62, 72, 82, 100, 110 | Livestock grazing and forestry incl. wood fuel |
| Pasture | Pasture layer from *HYDE* | Livestock grazing |
| Rangeland | Rangeland layer from *HYDE* | Livestock grazing |
| Other land herbaceous vegetation only | *ESA* layers 130, 140, 150, 151, 152, 153 | Livestock grazing |
| Other land herbaceous and woody vegetation | *ESA* layers 120, 121, 122, 180 | Livestock grazing and forestry incl. wood fuel |

### Regional coverage

Armenia

Afghanistan

Albania

Algeria

American Samoa

Andorra

Angola

Antigua and Barbuda

Argentina

Australia

Austria

Bahamas

Bahrain

Barbados

Belgium-Luxembourg

Bangladesh

Bermuda

Bhutan

Bolivia (Plurinational State of)

Botswana

Brazil

Aruba

Belize

Solomon Islands

Brunei Darussalam

Bulgaria

Myanmar

Burundi

Cameroon

Canada

Cabo Verde

Central African Republic

Sri Lanka

Chad

Chile

China, mainland

Colombia

Comoros

Congo

Costa Rica

Cuba

Cyprus

Czechoslovakia

Azerbaijan

Benin

Denmark

Dominica

Dominican Republic

Belarus

Ecuador

Egypt

El Salvador

Equatorial Guinea

Ethiopia PDR

Estonia

Faroe Islands

Falkland Islands (Malvinas)

Fiji

Finland

France

French Guiana

French Polynesia

Djibouti

Georgia

Gabon

Gambia

Germany

Bosnia and Herzegovina

Ghana

Kiribati

Greece

Greenland

Grenada

Guadeloupe

Guatemala

Guinea

Guyana

Haiti

Honduras

China, Hong Kong SAR

Hungary

Croatia

Iceland

India

Indonesia

Iran (Islamic Republic of)

Iraq

Ireland

Israel

Italy

Côte d'Ivoire

Kazakhstan

Jamaica

Japan

Jordan

Kyrgyzstan

Kenya

Cambodia

Democratic People's Republic of Korea

Republic of Korea

Kuwait

Latvia

Lao People's Democratic Republic

Lebanon

Lesotho

Liberia

Libya

Liechtenstein

Lithuania

China, Macao SAR

Madagascar

Malawi

Malaysia

Maldives

Mali

Malta

Martinique

Mauritania

Mauritius

Mexico

Mongolia

Montserrat

Morocco

Mozambique

Republic of Moldova

Namibia

Nepal

Netherlands

Netherlands Antilles (former)

New Caledonia

The former Yugoslav Republic of Macedonia

Vanuatu

New Zealand

Nicaragua

Niger

Nigeria

Norway

Pakistan

Panama

Czechia

Papua New Guinea

Paraguay

Peru

Philippines

Poland

Portugal

Guinea-Bissau

Timor-Leste

Puerto Rico

Eritrea

Qatar

Zimbabwe

RÃ©union

Romania

Rwanda

Russian Federation

Serbia and Montenegro

Saint Kitts and Nevis

Saint Lucia

Saint Pierre and Miquelon

Saint Vincent and the Grenadines

Sao Tome and Principe

Saudi Arabia

Senegal

Sierra Leone

Slovenia

Slovakia

Singapore

Somalia

South Africa

Spain

Western Sahara

Sudan (former)

Suriname

Tajikistan

Eswatini

Sweden

Switzerland

Syrian Arab Republic

Turkmenistan

China, Taiwan Province of

United Republic of Tanzania

Thailand

Togo

Tonga

Trinidad and Tobago

Oman

Tunisia

Turkey

Turks and Caicos Islands

United Arab Emirates

Uganda

USSR

United Kingdom

Ukraine

United States of America

Burkina Faso

Uruguay

Uzbekistan

Venezuela (Bolivarian Republic of)

Viet Nam

Ethiopia

United States Virgin Islands

Samoa

Yugoslav SFR

Yemen

Democratic Republic of the Congo

Zambia

Belgium

Luxembourg

Anguilla

Svalbard and Jan Mayen Islands

Serbia

Montenegro

Sudan

South Sudan

## Calculation steps

### Closed-budget land use maps

In order to construct a closed budget map global dataset we sort the individual maps by data reliability for each layer. Then, each layer is added to the sum of the previous layers in a step-wise approach. The values of the current layer are capped if the sum of this layer plus all previous layers exceeded the total available land area in a given pixels. The prioritization of the different layers follows the order of layers in Table 1 with highest priority for infrastructure land.

For the infrastructure layer, we performed an additional adjustment following Erb et al. (2007).To reflect the area requirement of small-scale rural infrastructure, we added 5% of the cropland area in each pixel as infrastructure land.

The two wilderness layers were constructed as follows: within the “forest zone” as specified by Potapov et al. (2017) we had estimates from both used data sources. For the human footprint data we considered areas with an index of 0 as wilderness (Venter et al. 2016). Following a conservative approach, we classify only areas identified in both products as wilderness as core wilderness areas. Areas identified as wilderness in only one of the products were classified as peripheral wilderness areas where light use is occurring. Outside the “forest zone” all values identified as wilderness in the human footprint dataset are considered core wilderness areas.

Certain data layers were not available for all years in the time series. In this case, we interpolated values linearly between available years and kept values constant after and before the last and first available year, respectively.

### Finer split between sectors

The following adjustments were performed at the national level, as the input data are available at this level only. The resulting dataset is available as table data but not as spatially explicit maps.

We split cropland into 21 sub-sectors based on information from FAOSTAT. Areas actually planted to crops in a given year were accounted for based on the amount of harvested area statistics from FAOSTAT’s crops domain. For this, we grouped individual crops into the EXIOBASE sector classification from Stadler et al. (2018). If harvested area was larger than cropland area (in case of multi-cropping) it was reduced proportionally for the different sectors to match available cropland area. If harvested area was smaller than cropland area, the difference between the two was considered fallow area. We attributed half of the fallow area proportionally to the different crop sectors and the other half to the primary livestock sectors. The rationale for this choice is that FAOSTAT recently removed its information on dedicated fodder crops (e.g. maize for silage, fodder legumes) and we assume part of the “fallow” are planted to fodder crops. Also, is not uncommon for livestock to graze on fallow land in many countries.

The split between the different livestock sectors was performed based on information on the production of livestock products in a given country and a generic weighing key that reflects conversion efficiencies and roughage share in feed of for five different livestock products. Based on literature (e.g., Smil, 2002), the following weighing factors were applied for fallow area (here also a certain share of feed going to poultry and pigs, groups that usually do not feed on roughage, was assumed): pig meat 2, milk 1, beef 20, sheep and goat meat 10, poultry 1. For the grazing sectors, only ruminant products were considered, resulting in the following weighing scheme: milk 1, beef 20, sheep and goat meat 10.

The allocation of grazing lands not classified as pasture or rangeland (the sum of the two corresponds to the FAOSTAT category *permanent pastures and meadows)* follows the procedure outlined in Stadler et al. (2018). In countries that were considered advanced economies according to the international monetary fund (see Stadler et al 2018), land use in these categories land is allocated to the primary livestock sectors, following the split outlined above. In all other countries land use is allocated to final household demand (subsistence grazing). On open forests and other lands with woody elements, we mainly assume wood fuel extraction as forestry activity. The allocation of these lands to either the forestry sector or to final household demand follows the procedure described in Stadler et al. (2018).

Table 2 shows the resulting classification of land use types, their attribution to EXIOBASE sectors and the global totals for each sector for the years 1995 and 2015 and the change between these two years. These final data are available, following this classification, for XXX countries, from 1995 to 2015.

Table S 13 land use categories and their attribution to EXIOBASE sectors along with global values for 1995 and 2015 and the change between the two years.

| **Land use category** | **EXIOBASE**  **sector** | **1995**  **[‘000 km²]** | **2015**  **[‘000 km²]** | **2015-1995**  **[‘000 km²]** |
| --- | --- | --- | --- | --- |
| Cropland - cropped area - Paddy rice | p01.a | 1,344 | 1,352 | 8 |
| Cropland - fallowed area - Paddy rice | p01.a | 59 | 39 | -20 |
| Cropland - cropped area - Wheat | p01.b | 2,099 | 2,106 | 8 |
| Cropland - fallowed area - Wheat | p01.b | 589 | 488 | -101 |
| Cropland - cropped area - Cereal grains nec | p01.c | 3,110 | 3,083 | -27 |
| Cropland - fallowed area - Cereal grains nec | p01.c | 699 | 484 | -215 |
| Cropland - cropped area - Vegetables, fruit, nuts | p01.d | 2,015 | 2,443 | 429 |
| Cropland - fallowed area - Vegetables, fruit, nuts | p01.d | 290 | 227 | -63 |
| Cropland - cropped area - Oil seeds | p01.e | 1,787 | 2,674 | 887 |
| Cropland - fallowed area - Oil seeds | p01.e | 323 | 363 | 40 |
| Cropland - cropped area - Sugar cane, sugar beet | p01.f | 256 | 293 | 37 |
| Cropland - fallowed area - Sugar cane, sugar beet | p01.f | 49 | 36 | -13 |
| Cropland - cropped area - Plant-based fibers | p01.g | 248 | 207 | -41 |
| Cropland - fallowed area - Plant-based fibers | p01.g | 39 | 17 | -22 |
| Cropland - cropped area - Crops nec | p01.h | 333 | 422 | 89 |
| Cropland - fallowed area - Crops nec | p01.h | 31 | 23 | -8 |
| Cropland - fallowed area - Cattle | p01.i | 1,235 | 893 | -342 |
| Grazing land - open forests - Cattle | p01.i | 511 | 528 | 16 |
| Grazing land - other used land - Cattle | p01.i | 1,084 | 1,246 | 163 |
| Grazing land - pastures - Cattle | p01.i | 3,288 | 3,075 | -213 |
| Grazing land - rangeland - Cattle | p01.i | 10,705 | 10,396 | -309 |
| Cropland - fallowed area - Pigs | p01.j | 87 | 94 | 7 |
| Cropland - fallowed area - Poultry | p01.k | 54 | 79 | 25 |
| Cropland - fallowed area - Meat animals nec | p01.l | 134 | 103 | -31 |
| Grazing land - open forests - Meat animals nec | p01.l | 54 | 43 | -10 |
| Grazing land - other used land - Meat animals nec | p01.l | 121 | 113 | -7 |
| Grazing land - pastures - Meat animals nec | p01.l | 547 | 539 | -9 |
| Grazing land - rangeland - Meat animals nec | p01.l | 1,960 | 1,860 | -100 |
| Cropland - fallowed area - Raw milk | p01.n | 569 | 510 | -60 |
| Grazing land - open forests - Raw milk | p01.n | 223 | 254 | 31 |
| Grazing land - other used land - Raw milk | p01.n | 390 | 475 | 85 |
| Grazing land - pastures - Raw milk | p01.n | 1,168 | 1,389 | 221 |
| Grazing land - rangeland - Raw milk | p01.n | 3,532 | 3,988 | 456 |
| Forestry incl. wood fuel - closed forests | p02 | 20,507 | 20,758 | 251 |
| Forestry incl. wood fuel - open forests | p02 | 1,341 | 1,979 | 639 |
| Forestry incl. wood fuel - other used land | p02 | 680 | 946 | 266 |
| Forestry incl. wood fuel - open forests | y01 | 4,824 | 4,387 | -437 |
| Forestry incl. wood fuel - other used land | y01 | 2,573 | 2,323 | -250 |
| Grazing land - open forests - final demand | y01 | 5,376 | 5,541 | 165 |
| Grazing land - other used land | y01 | 4,640 | 4,705 | 64 |
| Infrastructure land | y01 | 1,174 | 1,380 | 205 |
| Marginal use - wilderness periphery | y01 | 10,016 | 9,976 | -40 |
| Unused land - unproductive | - | 21,399 | 21,140 | -259 |
| Unused land - wilderness core | - | 20,342 | 18,825 | -1,516 |

# Emissions embodied in bilateral trade (EEBT)

The Emissions embodied in bilateral trade (EEBT) approach was used to calculate land use impacts in this paper. the equation for calculating impacts in an MRIO is the starting point:

|  | $F=SLY$ | (S1) |
| --- | --- | --- |

$F$ Total impacts (in our case land use)

$S$ Impact intensity per monetary unit

$L$ The Leontief inverse

$Y$ Demand

Following Peters and Hertwich (2008), we split demand $Y$ into a domestic and a traded component, so that the production-based ($pb$) emissions from a specific region $r$ becomes

|  | $F_{r}^{pb}=S_{r}L_{rr}(Y_{rr}*\sum_{s} e_{rs})$ | (S2) |
| --- | --- | --- |

$e_{rs}$ Exports from region $r$ to region $s$.

$pb$ Production-based emissions

To obtain the consumption-based impacts ($cb$) for region r, we add the impacts from consumption of imported goods to the consumption of domestically produced goods:

|  | $F_{r}^{cb}=S_{r}L_{rr}Y_{rr}+\sum_{s} {(S}_{s}L_{ss}*e_{sr})$ | (S3) |
| --- | --- | --- |

$e_{sr}$ Imports from region $s$ to region $r$

# Total land use results for 214 regions

Table S 14: Land area use from production, consumption, exports as share of production, imports as share of consumption and the balance of land area embodied in trade (BLET) for EXIOBASE 3rx for 214 regions.

| **Country Name** | **Production (km2)** | **Consumption (km2)** | **exports %** | **imports %** | **BLET %** |
| --- | --- | --- | --- | --- | --- |
| Afghanistan | 395000 | 409000 | 1.1 | 4.6 | -3.4 |
| Albania | 27900 | 30700 | 12.5 | 20.4 | -8.0 |
| Algeria | 229000 | 280000 | 13.5 | 29.3 | -15.8 |
| Andorra | 354 | 807 | 4.7 | 58.2 | -53.5 |
| Angola | 1220000 | 972000 | 25.8 | 7.1 | 18.8 |
| Anguilla | 82 | 195 | 0.1 | 58.1 | -58.0 |
| Antigua and Barbuda | 441 | 1030 | 3.7 | 58.8 | -55.2 |
| Argentina | 2500000 | 1920000 | 25.6 | 2.9 | 22.7 |
| Armenia | 27900 | 34200 | 10.1 | 26.8 | -16.7 |
| Aruba | 80 | 1500 | 0.0 | 94.7 | -94.6 |
| Australia | 4870000 | 1800000 | 64.9 | 5.1 | 59.8 |
| Austria | 80300 | 119000 | 48.6 | 65.4 | -16.8 |
| Azerbaijan | 81300 | 134000 | 12.4 | 46.8 | -34.4 |
| Bahamas | 9160 | 11800 | 0.5 | 22.6 | -22.1 |
| Bahrain | 416 | 12400 | 13.6 | 97.1 | -83.5 |
| Bangladesh | 137000 | 197000 | 16.8 | 42.2 | -25.4 |
| Barbados | 441 | 2400 | 2.2 | 82.0 | -79.8 |
| Belarus | 204000 | 156000 | 41.4 | 23.3 | 18.2 |
| Belgium | 30600 | 227000 | 66.6 | 95.5 | -28.9 |
| Belize | 21300 | 12700 | 44.7 | 7.4 | 37.4 |
| Benin | 115000 | 100000 | 23.5 | 12.6 | 10.9 |
| Bermuda | 3 | 139 | 0.2 | 97.8 | -97.7 |
| Bhutan | 37400 | 31900 | 16.4 | 1.9 | 14.6 |
| Bolivia | 930000 | 690000 | 28.5 | 3.5 | 24.9 |
| Bosnia and Herzegovina | 51100 | 48900 | 20.2 | 16.6 | 3.6 |
| Botswana | 455000 | 426000 | 13.9 | 8.2 | 5.8 |
| Brazil | 6950000 | 5810000 | 19.4 | 3.5 | 15.9 |
| British Virgin Islands | 145 | 203 | 20.5 | 43.0 | -22.5 |
| Brunei Darussalam | 6290 | 10600 | 17.0 | 50.9 | -33.9 |
| Bulgaria | 110000 | 75100 | 44.7 | 19.4 | 25.3 |
| Burkina Faso | 274000 | 241000 | 14.9 | 3.4 | 11.6 |
| Burundi | 25000 | 26100 | 6.4 | 10.6 | -4.2 |
| Cabo Verde | 2910 | 3610 | 4.9 | 23.3 | -18.5 |
| Cambodia | 182000 | 136000 | 27.8 | 3.2 | 24.6 |
| Cameroon | 465000 | 343000 | 28.8 | 3.6 | 25.2 |
| Canada | 3410000 | 2700000 | 29.7 | 11.0 | 18.7 |
| Cayman Islands | 243 | 477 | 3.1 | 50.7 | -47.6 |
| Central African Republic | 620000 | 484000 | 22.4 | 0.6 | 21.8 |
| Chad | 633000 | 523000 | 17.7 | 0.4 | 17.3 |
| Chile | 467000 | 487000 | 17.5 | 20.9 | -3.4 |
| China | 6990000 | 12300000 | 15.8 | 51.9 | -36.2 |
| Hong Kong | 863 | 306000 | 8.0 | 99.7 | -91.7 |
| Macao | 23 | 10400 | 5.0 | 99.8 | -94.8 |
| Colombia | 907000 | 840000 | 14.7 | 7.9 | 6.8 |
| Comoros |  |  |  |  |  |
| Congo Republic | 334000 | 270000 | 22.1 | 3.7 | 18.4 |
| Cook Islands | 219 | 189 | 42.4 | 33.2 | 9.2 |
| Costa Rica | 50600 | 47100 | 49.0 | 45.3 | 3.7 |
| Croatia | 54800 | 53700 | 20.2 | 18.6 | 1.6 |
| Cuba | 106000 | 124000 | 9.5 | 22.6 | -13.1 |
| Curacao | 369 | 3510 | 8.2 | 90.3 | -82.1 |
| Cyprus | 9000 | 9980 | 38.6 | 44.7 | -6.0 |
| Czech Republic | 78800 | 88000 | 49.1 | 54.5 | -5.4 |
| Cote d'Ivoire | 321000 | 257000 | 26.9 | 8.7 | 18.1 |
| North Korea | 125000 | 119000 | 5.1 | 0.6 | 4.5 |
| DR Congo | 2300000 | 1820000 | 22.8 | 2.5 | 20.3 |
| Denmark | 43300 | 72300 | 56.0 | 73.6 | -17.6 |
| Djibouti | 2760 | 6050 | 16.3 | 61.9 | -45.6 |
| Dominica | 763 | 919 | 9.9 | 25.1 | -15.3 |
| Dominican Republic | 47800 | 75600 | 16.5 | 47.2 | -30.7 |
| Ecuador | 224000 | 145000 | 46.4 | 16.9 | 29.5 |
| Egypt | 65300 | 319000 | 12.9 | 82.2 | -69.2 |
| El Salvador | 19800 | 26200 | 37.6 | 52.8 | -15.2 |
| Equatorial Guinea | 26900 | 7160 | 83.4 | 37.7 | 45.7 |
| Eritrea | 73300 | 71000 | 4.5 | 1.3 | 3.2 |
| Estonia | 43200 | 21300 | 77.3 | 53.9 | 23.3 |
| Ethiopia | 1050000 | 1010000 | 5.8 | 1.9 | 3.9 |
| Fiji | 16800 | 16000 | 27.7 | 24.3 | 3.4 |
| Finland | 284000 | 467000 | 35.5 | 60.7 | -25.2 |
| Netherlands Antilles |  |  |  |  |  |
| France | 588000 | 837000 | 32.8 | 52.8 | -20.0 |
| French Polynesia | 255 | 2880 | 11.1 | 92.1 | -81.1 |
| Gabon | 263000 | 184000 | 31.3 | 1.7 | 29.6 |
| Gambia | 9910 | 8920 | 16.9 | 7.6 | 9.2 |
| Georgia | 68400 | 67300 | 23.2 | 22.1 | 1.2 |
| Germany | 355000 | 796000 | 37.3 | 72.0 | -34.7 |
| Ghana | 234000 | 182000 | 31.9 | 12.6 | 19.3 |
| Greece | 126000 | 134000 | 32.6 | 36.4 | -3.7 |
| Greenland | 35600 | 35900 | 0.1 | 0.7 | -0.7 |
| Grenada | 321 | 729 | 7.4 | 59.2 | -51.8 |
| Guatemala | 109000 | 102000 | 26.4 | 21.0 | 5.3 |
| Guinea | 245000 | 235000 | 6.7 | 2.8 | 3.9 |
| Guinea-Bissau | 32200 | 27700 | 16.5 | 3.0 | 13.5 |
| Guyana | 132000 | 103000 | 23.1 | 1.6 | 21.5 |
| Haiti |  |  |  |  |  |
| Honduras | 110000 | 68500 | 50.8 | 21.0 | 29.8 |
| Hungary | 92100 | 73700 | 52.8 | 41.1 | 11.8 |
| Iceland | 46800 | 30900 | 37.8 | 5.9 | 32.0 |
| India | 3070000 | 3390000 | 9.4 | 18.1 | -8.7 |
| Indonesia | 1810000 | 2160000 | 16.1 | 29.6 | -13.6 |
| Iran | 560000 | 636000 | 25.5 | 34.5 | -9.0 |
| Iraq | 115000 | 149000 | 13.9 | 33.8 | -19.9 |
| Ireland | 70200 | 63300 | 75.2 | 72.5 | 2.7 |
| Israel | 13900 | 126000 | 35.1 | 92.8 | -57.7 |
| Italy | 290000 | 571000 | 24.3 | 61.5 | -37.2 |
| Jamaica | 11000 | 19400 | 12.8 | 50.8 | -38.0 |
| Japan | 410000 | 1220000 | 5.0 | 68.2 | -63.1 |
| Jordan | 8130 | 42900 | 49.3 | 90.4 | -41.1 |
| Kazakhstan | 2330000 | 2020000 | 16.6 | 4.0 | 12.7 |
| Kenya | 544000 | 565000 | 7.4 | 10.9 | -3.5 |
| Kiribati | 340 | 443 | 28.6 | 45.2 | -16.6 |
| Kosovo | 11100 | 12400 | 6.5 | 16.4 | -9.9 |
| Kuwait | 1550 | 102000 | 26.4 | 98.9 | -72.4 |
| Kyrgyz Republic | 168000 | 173000 | 5.7 | 8.5 | -2.8 |
| Laos | 230000 | 179000 | 22.5 | 0.7 | 21.8 |
| Latvia | 64200 | 43300 | 79.5 | 69.6 | 9.9 |
| Lebanon | 10300 | 86200 | 26.1 | 91.2 | -65.0 |
| Lesotho | 30600 | 25000 | 26.9 | 10.4 | 16.4 |
| Liberia | 94000 | 73800 | 22.1 | 0.8 | 21.3 |
| Libya | 60800 | 95800 | 16.0 | 46.6 | -30.7 |
| Liechtenstein |  |  |  |  |  |
| Lithuania | 64100 | 57500 | 57.0 | 52.0 | 5.0 |
| Luxembourg | 2500 | 26800 | 75.6 | 97.7 | -22.1 |
| Madagascar | 589000 | 500000 | 15.6 | 0.6 | 15.0 |
| Malawi | 96500 | 86300 | 26.2 | 17.5 | 8.7 |
| Malaysia | 332000 | 402000 | 35.0 | 46.4 | -11.4 |
| Maldives | 52 | 1270 | 19.2 | 96.7 | -77.4 |
| Mali | 544000 | 483000 | 12.9 | 2.0 | 10.9 |
| Malta | 238 | 3800 | 26.0 | 95.4 | -69.4 |
| Marshall Islands | 181 | 188 | 0.7 | 4.5 | -3.8 |
| Mauritania | 195000 | 164000 | 18.1 | 2.4 | 15.7 |
| Mauritius | 790 | 53200 | 34.9 | 99.0 | -64.1 |
| Mexico | 1910000 | 1710000 | 26.2 | 17.5 | 8.8 |
| Micronesia, Fed. Sts. | 702 | 949 | 4.3 | 29.2 | -24.9 |
| Monaco | 1720 | 2840 | 4.2 | 42.1 | -37.9 |
| Mongolia | 838000 | 590000 | 30.3 | 1.0 | 29.3 |
| Montenegro | 13500 | 14600 | 12.2 | 18.3 | -6.1 |
| Montserrat | 93 | 124 | 2.5 | 27.0 | -24.6 |
| Morocco | 229000 | 239000 | 21.0 | 24.1 | -3.2 |
| Mozambique | 772000 | 620000 | 26.0 | 7.8 | 18.2 |
| Myanmar | 665000 | 537000 | 20.7 | 1.8 | 19.0 |
| Namibia | 563000 | 427000 | 25.7 | 2.0 | 23.7 |
| Nauru | 20 | 530 | 0.3 | 96.2 | -96.0 |
| Nepal | 139000 | 130000 | 12.8 | 7.0 | 5.8 |
| Netherlands | 35700 | 336000 | 69.9 | 96.8 | -26.9 |
| New Caledonia | 18600 | 21200 | 6.6 | 17.8 | -11.2 |
| New Zealand | 240000 | 179000 | 45.9 | 27.3 | 18.6 |
| Nicaragua | 118000 | 65200 | 51.5 | 11.9 | 39.6 |
| Niger | 360000 | 285000 | 23.7 | 3.8 | 19.9 |
| Nigeria | 904000 | 836000 | 20.9 | 14.5 | 6.4 |
| Norway | 262000 | 209000 | 47.6 | 34.3 | 13.3 |
| Oman | 24600 | 37100 | 37.8 | 58.8 | -21.0 |
| Pakistan | 624000 | 639000 | 10.9 | 13.1 | -2.2 |
| Palau | 461 | 470 | 63.7 | 64.4 | -0.6 |
| Panama | 74200 | 67500 | 30.5 | 23.6 | 6.9 |
| Papua New Guinea | 471000 | 318000 | 33.8 | 2.1 | 31.8 |
| Paraguay | 393000 | 244000 | 43.9 | 9.6 | 34.3 |
| Peru | 938000 | 892000 | 15.3 | 11.0 | 4.3 |
| Philippines | 299000 | 382000 | 14.0 | 32.7 | -18.7 |
| Poland | 310000 | 310000 | 36.8 | 36.8 | 0.0 |
| Portugal | 88700 | 183000 | 37.5 | 69.7 | -32.2 |
| Puerto Rico | 8550 | 97000 | 15.3 | 92.5 | -77.2 |
| Qatar | 1320 | 79500 | 16.4 | 98.6 | -82.2 |
| South Korea | 105000 | 719000 | 11.7 | 87.1 | -75.4 |
| Moldova | 33500 | 23100 | 55.4 | 35.3 | 20.2 |
| Romania | 236000 | 183000 | 36.2 | 17.7 | 18.5 |
| Russia | 10200000 | 7110000 | 33.6 | 4.9 | 28.7 |
| Rwanda | 24200 | 32900 | 12.7 | 35.8 | -23.1 |
| St. Kitts and Nevis | 201 | 432 | 5.4 | 55.9 | -50.5 |
| St. Lucia | 612 | 994 | 11.3 | 45.4 | -34.0 |
| Samoa | 2660 | 3080 | 5.3 | 18.4 | -13.2 |
| San Marino | 59 | 561 | 56.4 | 95.4 | -39.0 |
| Sao Tome and Principe | 1080 | 1110 | 18.1 | 20.5 | -2.5 |
| Saudi Arabia | 122000 | 296000 | 45.8 | 77.6 | -31.8 |
| Senegal | 197000 | 186000 | 10.1 | 4.9 | 5.2 |
| Serbia | 88100 | 73500 | 32.1 | 18.6 | 13.4 |
| Seychelles | 467 | 3760 | 9.6 | 88.8 | -79.2 |
| Sierra Leone | 71600 | 53900 | 28.0 | 4.3 | 23.7 |
| Singapore | 448 | 154000 | 0.5 | 99.7 | -99.2 |
| Sint Maarten | 29 | 1390 | 33.1 | 98.6 | -65.5 |
| Slovakia | 48900 | 37600 | 69.4 | 60.2 | 9.2 |
| Slovenia | 20300 | 24400 | 49.7 | 58.0 | -8.4 |
| Solomon Islands | 28500 | 19300 | 40.4 | 11.7 | 28.7 |
| Somalia | 582000 | 573000 | 1.6 | 0.0 | 1.5 |
| South Africa | 1190000 | 952000 | 28.7 | 10.5 | 18.2 |
| South Sudan |  |  |  |  |  |
| Spain | 499000 | 517000 | 39.9 | 42.0 | -2.1 |
| Sri Lanka | 65500 | 80700 | 20.9 | 35.8 | -14.9 |
| St. Vincent and the Grenadines | 345 | 805 | 3.1 | 58.5 | -55.4 |
| Palestine | 551 | 6630 | 25.4 | 93.8 | -68.4 |
| Sudan |  |  |  |  |  |
| Suriname | 51600 | 35600 | 33.0 | 2.9 | 30.1 |
| Swaziland | 16900 | 15300 | 25.4 | 17.2 | 8.2 |
| Sweden | 394000 | 424000 | 38.5 | 42.9 | -4.5 |
| Switzerland | 36000 | 72600 | 49.4 | 74.9 | -25.5 |
| Syria | 77700 | 66500 | 40.3 | 30.2 | 10.1 |
| Macedonia | 24700 | 24000 | 22.3 | 20.1 | 2.2 |
| Tajikistan | 86500 | 105000 | 3.8 | 20.9 | -17.1 |
| Thailand | 513000 | 485000 | 35.0 | 31.2 | 3.8 |
| Timor-Leste | 15500 | 11000 | 31.6 | 4.3 | 27.3 |
| Togo | 57500 | 46500 | 23.0 | 4.9 | 18.1 |
| Tonga | 322 | 449 | 34.7 | 53.2 | -18.6 |
| Trinidad and Tobago | 5330 | 19900 | 11.5 | 76.3 | -64.8 |
| Tunisia | 74000 | 80100 | 28.5 | 33.9 | -5.4 |
| Turkey | 761000 | 971000 | 13.1 | 31.9 | -18.8 |
| Turkmenistan | 144000 | 91700 | 51.4 | 23.5 | 27.9 |
| Turks and Caicos Islands | 229 | 1550 | 2.6 | 85.6 | -83.1 |
| Tuvalu | 19 | 36 | 1.5 | 47.6 | -46.2 |
| Tanzania | 888000 | 757000 | 17.7 | 3.5 | 14.2 |
| Uganda | 204000 | 183000 | 18.9 | 9.6 | 9.3 |
| Ukraine | 585000 | 293000 | 56.8 | 13.7 | 43.1 |
| United Arab Emirates | 9670 | 207000 | 24.9 | 96.5 | -71.6 |
| United Kingdom | 248000 | 515000 | 22.0 | 62.3 | -40.4 |
| United States | 7740000 | 7840000 | 23.9 | 24.8 | -1.0 |
| Uruguay | 174000 | 117000 | 61.4 | 42.6 | 18.8 |
| Uzbekistan | 190000 | 204000 | 20.7 | 26.3 | -5.6 |
| Vanuatu | 12000 | 7340 | 41.1 | 3.7 | 37.4 |
| Venezuela | 669000 | 984000 | 3.7 | 34.5 | -30.8 |
| Vietnam | 325000 | 609000 | 34.8 | 65.2 | -30.4 |
| Yemen | 97800 | 117000 | 16.0 | 29.7 | -13.7 |
| Zambia | 744000 | 551000 | 30.0 | 5.5 | 24.5 |
| Zanzibar | 2310 | 2640 | 16.5 | 26.8 | -10.4 |
| Zimbabwe | 389000 | 407000 | 7.6 | 11.6 | -4.1 |
| Taiwan | 35800 | 1230000 | 48.6 | 98.5 | -49.9 |
| **Total** | **90300000** | **90300000** | **25.8** | **25.8** | **0.0** |

# References

Erb, K.-H., Gaube, V., Krausmann, F., Plutzar, C., Bondeau, A., Haberl, H., 2007. A comprehensive global 5 min resolution land-use data set for the year 2000 consistent with national census data. J. Land Use Sci. 2, 191–224. https://doi.org/10.1080/17474230701622981

Klein Goldewijk, K., Beusen, A., Doelman, J., Stehfest, E., 2017. Anthropogenic land use estimates for the Holocene – HYDE 3.2. Earth Syst. Sci. Data 9, 927–953. https://doi.org/10.5194/essd-9-927-2017

Potapov, P., Hansen, M.C., Laestadius, L., Turubanova, S., Yaroshenko, A., Thies, C., Smith, W., Zhuravleva, I., Komarova, A., Minnemeyer, S., Esipova, E., 2017. The last frontiers of wilderness: Tracking loss of intact forest landscapes from 2000 to 2013. Sci. Adv. 3, e1600821. https://doi.org/10.1126/sciadv.1600821

Smil, V., 2002. Worldwide transformation of diets, burdens of meat production and opportunities for novel food proteins. Enzyme Microb. Technol. 30, 305–311. https://doi.org/10.1016/S0141-0229(01)00504-X

Stadler, K., Wood, R., Bulavskaya, T., Södersten, C.-J., Simas, M., Schmidt, S., Usubiaga, A., Acosta‐Fernández, J., Kuenen, J., Bruckner, M., Giljum, S., Lutter, S., Merciai, S., Schmidt, J.H., Theurl, M.C., Plutzar, C., Kastner, T., Eisenmenger, N., Erb, K.-H., Koning, A. de, Tukker, A., 2018. EXIOBASE 3: Developing a Time Series of Detailed Environmentally Extended Multi-Regional Input-Output Tables. J. Ind. Ecol. 22, 502–515. https://doi.org/10.1111/jiec.12715

Venter, O., Sanderson, E.W., Magrach, A., Allan, J.R., Beher, J., Jones, K.R., Possingham, H.P., Laurance, W.F., Wood, P., Fekete, B.M., Levy, M.A., Watson, J.E.M., 2016. Sixteen years of change in the global terrestrial human footprint and implications for biodiversity conservation. Nat. Commun. 7, 12558. https://doi.org/10.1038/ncomms12558

FAOSTAT. 2014. *FAOSTAT—Prodstat; Food and Agriculture Organization of the United Nations* [Online]. FAO Statistics Division. Available: <http://faostat.fao.org/site/567/DesktopDefault.aspx#ancor> [Accessed 25 February 2016].

GAULIER, G. & ZIGNAGO, S. 2010. Baci: international trade database at the product-level (the 1994-2007 version).

INTERNATIONAL ENERGY AGENCY. 2018. *IEA Data Services* [Online]. International Energy Agency,. Available: <http://data.iea.org/> [Accessed September 24 2018].

PETERS, G. P. & HERTWICH, E. G. 2008. CO2 embodied in international trade with implications for global climate policy. ACS Publications.

STADLER, K., WOOD, R., BULAVSKAYA, T., SÖDERSTEN, C. J., SIMAS, M., SCHMIDT, S., USUBIAGA, A., ACOSTA‐FERNÁNDEZ, J., KUENEN, J. & BRUCKNER, M. 2018. EXIOBASE 3: Developing a Time Series of Detailed Environmentally Extended Multi‐Regional Input‐Output Tables. *Journal of Industrial Ecology*.

UNITED NATIONS. 2018. *National Accounts Main Aggregates Database* [Online]. Available: <https://unstats.un.org/unsd/snaama/dnllist.asp> [Accessed September 24 2018].

1. UN National Accounts of Main Aggegates UNITED NATIONS. 2018. *National Accounts Main Aggregates Database* [Online]. Available: https://unstats.un.org/unsd/snaama/dnllist.asp [Accessed September 24 2018]. [↑](#footnote-ref-1)
2. For more details, see: STADLER, K., WOOD, R., BULAVSKAYA, T., SÖDERSTEN, C. J., SIMAS, M., SCHMIDT, S., USUBIAGA, A., ACOSTA‐FERNÁNDEZ, J., KUENEN, J. & BRUCKNER, M. 2018. EXIOBASE 3: Developing a Time Series of Detailed Environmentally Extended Multi‐Regional Input‐Output Tables. *Journal of Industrial Ecology*. [↑](#footnote-ref-2)
3. BACI –COMTRADE database GAULIER, G. & ZIGNAGO, S. 2010. Baci: international trade database at the product-level (the 1994-2007 version). [↑](#footnote-ref-3)
4. International Energy Agency Energy Balances INTERNATIONAL ENERGY AGENCY. 2018. *IEA Data Services* [Online]. International Energy Agency,. Available: http://data.iea.org/ [Accessed September 24 2018]. [↑](#footnote-ref-4)
5. FAOSTAT: FAOSTAT. 2014. *FAOSTAT—Prodstat; Food and Agriculture Organization of the United Nations* [Online]. FAO Statistics Division. Available: http://faostat.fao.org/site/567/DesktopDefault.aspx#ancor [Accessed 25 February 2016]. [↑](#footnote-ref-5)
